# Molecular Mechanisms of Reduced Nerve Toxicity by Titanium Dioxide Nanoparticles in the Phoxim-Exposed Brain of *Bombyx mori*

**DOI:** 10.1371/journal.pone.0101062

**Published:** 2014-06-27

**Authors:** Yi Xie, Binbin Wang, Fanchi Li, Lie Ma, Min Ni, Weide Shen, Fashui Hong, Bing Li

**Affiliations:** 1 School of Basic Medicine and Biological Sciences, Soochow University, Suzhou, Jiangsu, P.R. China; 2 National Engineering Laboratory for Modern Silk, Soochow University, Suzhou, Jiangsu, P.R. China; RMIT University, Australia

## Abstract

*Bombyx mori* (*B. mori*), silkworm, is one of the most important economic insects in the world, while phoxim, an organophosphorus (OP) pesticide, impact its economic benefits seriously. Phoxim exposure can damage the brain, fatbody, midgut and haemolymph of *B. mori*. However the metabolism of proteins and carbohydrates in phoxim-exposed *B. mori* can be improved by Titanium dioxide nanoparticles (TiO_2_ NPs). In this study, we explored whether TiO_2_ NPs treatment can reduce the phoxim-induced brain damage of the 5th larval instar of *B. mori*. We observed that TiO_2_ NPs pretreatments significantly reduced the mortality of phoxim-exposed larva and relieved severe brain damage and oxidative stress under phoxim exposure in the brain. The treatments also relieved the phoxim-induced increases in the contents of acetylcholine (Ach), glutamate (Glu) and nitric oxide (NO) and the phoxim-induced decreases in the contents of norepinephrine (NE), Dopamine (DA), and 5-hydroxytryptamine (5-HT), and reduced the inhibition of acetylcholinesterase (AChE), Na^+^/K^+^-ATPase, Ca^2+^-ATPase, and Ca^2+^/Mg^2+^-ATPase activities and the activation of total nitric oxide synthase (TNOS) in the brain. Furthermore, digital gene expression profile (DGE) analysis and real time quantitative PCR (qRT-PCR) assay revealed that TiO_2_ NPs pretreatment inhibited the up-regulated expression of *ace1, cytochrome c*, *caspase-9*, *caspase-3*, *Bm109* and down-regulated expression of *BmIap* caused by phoxim; these genes are involved in nerve conduction, oxidative stress and apoptosis. TiO_2_ NPs pretreatment also inhibited the down-regulated expression of *H^+^ transporting ATP synthase* and *vacuolar ATP synthase* under phoxim exposure, which are involved in ion transport and energy metabolism. These results indicate that TiO_2_ NPs pretreatment reduced the phoxim-induced nerve toxicity in the brain of *B. mori*.

## Introduction

Silkworm, *Bombyx mori* (*B. mori*, *Bombycidae: Lepidoptera*), is one of the most important economic insects in Asia, Africa, Europe and Latin America. *B. mori* has been domesticated for about 5,700 years in China, and it produces more than 80% of raw silk around the world [1]. However, *B. mori* is highly sensitive to adverse environmental conditions, especially pesticides. Every year, pesticide contamination causes as much as 30% of the reduction in raw silk production in China [2]. Due to its short growth cycle and pesticide sensitivity, *B. mori* has been a widely used model insect for pesticide toxicology studies. Phoxim is an efficient broad-spectrum organophosphorus (OP) pesticide, but its indiscriminate use has generated serious environmental problems.

Phoxim may trigger oxidative stress, which is mainly reflected in altered Malondialdehyde (MDA) content and Glutathione S-transferase (GST) activity in the fat body and midgut of *B. mori* [3]. Our previous study demonstrated that phoxim destroyed the carbohydrate and lipid metabolism in the haemolymph of *B. mori* [4]. Exposure of *B. mori* to phoxim also affected the activities of acetylcholinesterase (AChE) and detoxification enzymes, which play important roles in organophosphorus pesticide resistance and metabolism [5]. The catalytic substrate of AChE, Acetylcholine (ACh), is a chemical transmitter of cholinergic neurons that are exclusively in the central nervous system (CNS) of insects [6]. However, clear understanding of phoxim’s effects on the the brain of *B. mori* is still lacking. We hypothesized that nerve toxicity of phoxim in *B. mori* is associated with brain damages and gene expression profile alterations.

Titanium dioxide nanoparticles (TiO_2_ NPs) are widely used as whitening agents in paper, cosmetics, and food industries because of their whitening effects. TiO_2_ NPs may also be used for photocatalytic degradation of pesticide in water, soil, and air [7]–[9]. The growth of plants can be promoted by TiO_2_ NPs that improve their antioxidative capacity [10], [11]. Recently, it was reported that TiO_2_ NPs increased the cold-tolerance of *Chickpea* [12]. Our previous studies have shown that TiO_2_ NPs improve protein and carbohydrate metabolism to meet required energy demands and increase antioxidant capacity of midgut in *B. mori* exposed to phoxim [4], [13]. It was also found TiO_2_ NPs pretreatment decreased phoxim-induced toxicity to silkworms by greatly reducing the phoxim residue [14]. Therefore, we speculated that TiO_2_ NPs treatments may relieve phoxim-induced damage by modulating gene expression and enzymatic activities in the brain of *B. mori*.

Digital Gene Expression Profile (DGE) with massive parallel sequencing has been shown to have high sensitivity and reproducibility for transcriptome profiling [15]. DGE is based on new generation high-throughput sequencing technologies and high-performance computing analyses. Nowadays DGE has been widely used in biological, medical and pharmaceutical research [16]–[18].

In this study, we investigated the nerve toxicity of phoxim and the effects of TiO_2_ NPs in the brain of *B. mori*. To further explore the mechanisms of toxicity, we adopted DGE assay and real time quantitative PCR (qRT-PCR) to detect the alterations of genes participated in regulating neurotransmitter contents, oxidative stress and apoptosis. These findings may promote future mechanistic studies on the effects of TiO_2_ NPs on the toxicity of insecticides in *B. mori*.

## Results

### Body weight and Survival rate

**Figure 1:**
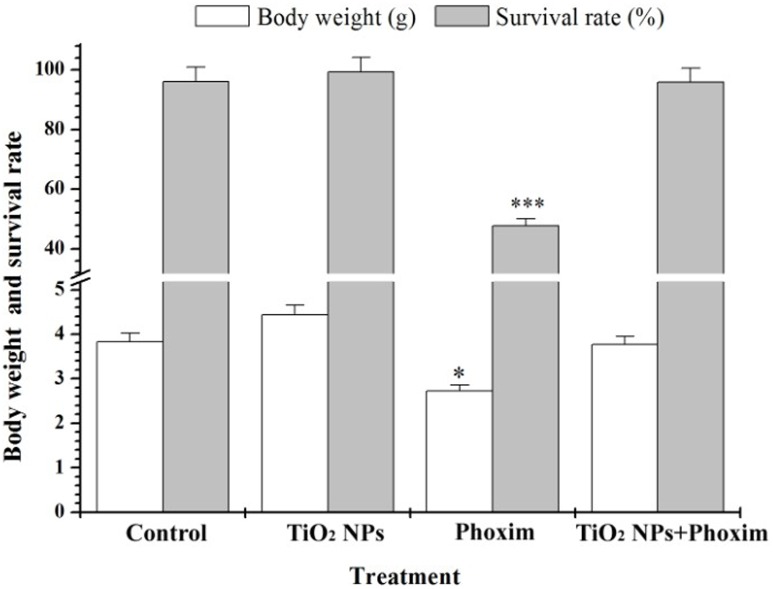
Effects of TiO_2_ NPs on body weight, survival of phoxim-exposed fifth-instar larvae. *P<0.05, and ***P<0.001. Values represent means ± SEM (*n* = 5).

We observed that the fifth-instar larvae appeared as gastric juice spit, head nystagmus, body distortion, body shrink, paralysis and other symptoms after 48 h of phoxim exposure. However, the larvae in the control group, the TiO_2_ NPs group, and the TiO_2_ NPs + phoxim group did not show such symptoms. As shown in Figure 1, phoxim exposure significantly decreased the body weight (*P*<0.05) and survival rate (*P*<0.001) of the larvae, while TiO_2_ NPs promoted their body weights and survival rate.

### Histopathological evaluation

**Figure 2:**
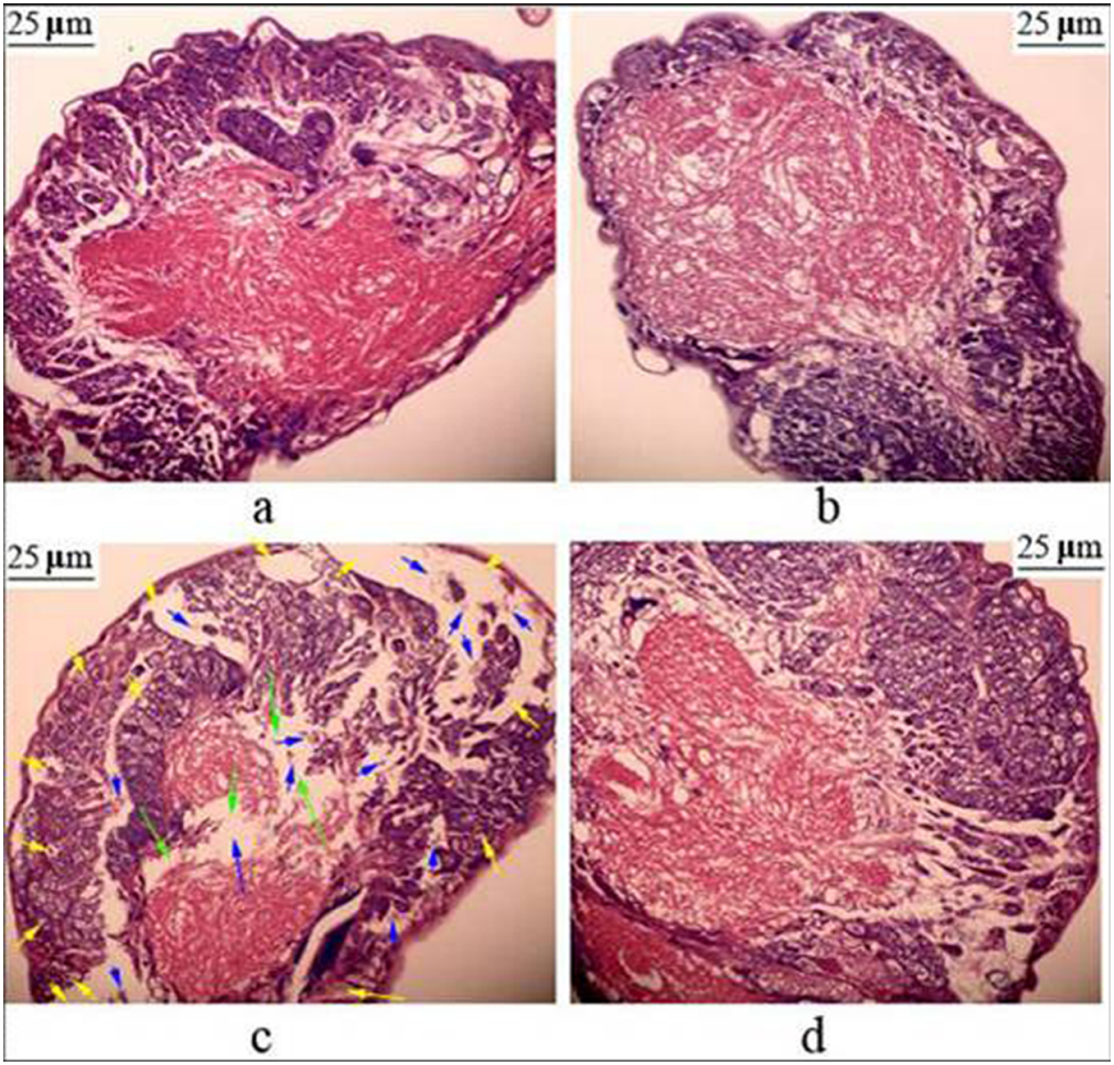
Histopathology of the brain tissue in fifth-instar larvae after phoxim exposure 48 h. (a) Control; (b) TiO_2_ NPs; (c) Phoxim; (d) TiO_2_ NPs + Phoxim. Green arrows indicate breakage of nerve fibers, yellow arrows show adipose degeneration, blue arrows indicate cell debris.

The brains of the larvae of both the control group (Fig. 2a) and the TiO_2_ NPs-treated group (Fig. 2b) had normal morphology. In the phoxim-exposed group, we observed widespread gaps among plasma membrane, breakage of nerve fibers, protein aggregation, adipose degeneration, and cell debris (Fig. 2c). However, the TiO_2_ NPs + phoxim-treated group did not show such pathological changes (Fig. 2d). It demonstrated that phoxim exposure caused brain damages, while TiO_2_ NPs treatments were able to reduce such damages.

### Brain ultrastructure evaluation

**Figure 3:**
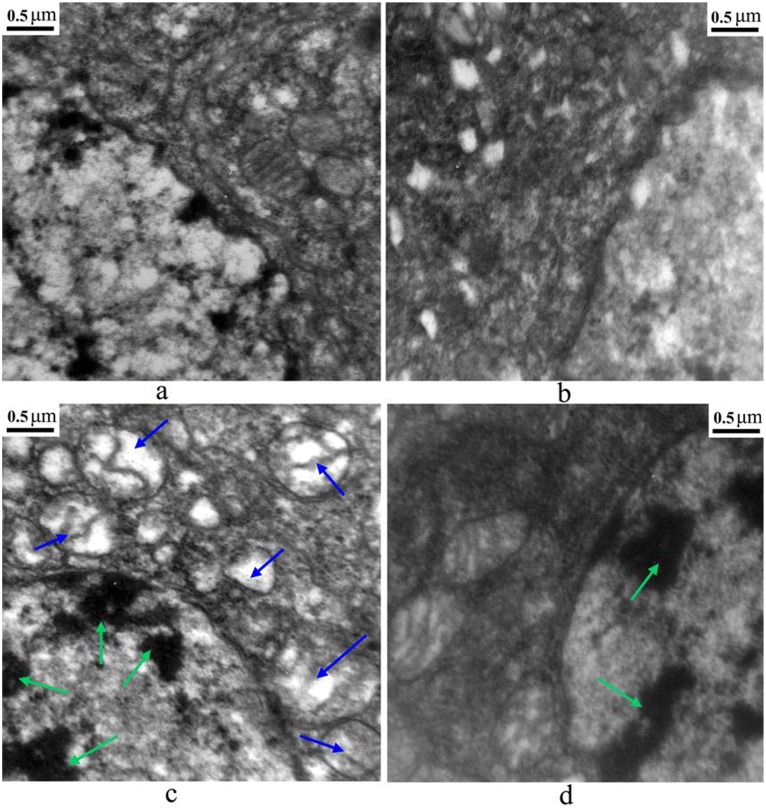
Ultrastructure of the brain tissue in fifth-instar larvae after phoxim exposure 48 h. (a) Control; (b) TiO_2_ NPs; (c) Phoxim; (d) TiO_2_ NPs + Phoxim. Green arrows indicate karyopyknosis and chromatin marginalization, blue arrows show mitochondria swelling and became deformed, crest broken.

As shown in Figure 3, the ultrastructure of cells in the control group and the TiO_2_ NPs group was normal with well distributed chromatin and integral mitochondria crista (Fig. 3a, 3b), compared with karyopyknosis, chromatin marginalization, and mitochondria swelling in the phoxim exposure group (Fig. 3c) at 48 h after phoxim exposure. However, only chromatin marginalization was observed in the TiO_2_ NPs + phoxim group (Fig. 3d), indicating that TiO_2_ NPs reduced the damage in *B. mori* brain cells caused by phoxim exposure.

### Neurotransmitter contents and enzyme activities in the brain

**Figure 4:**
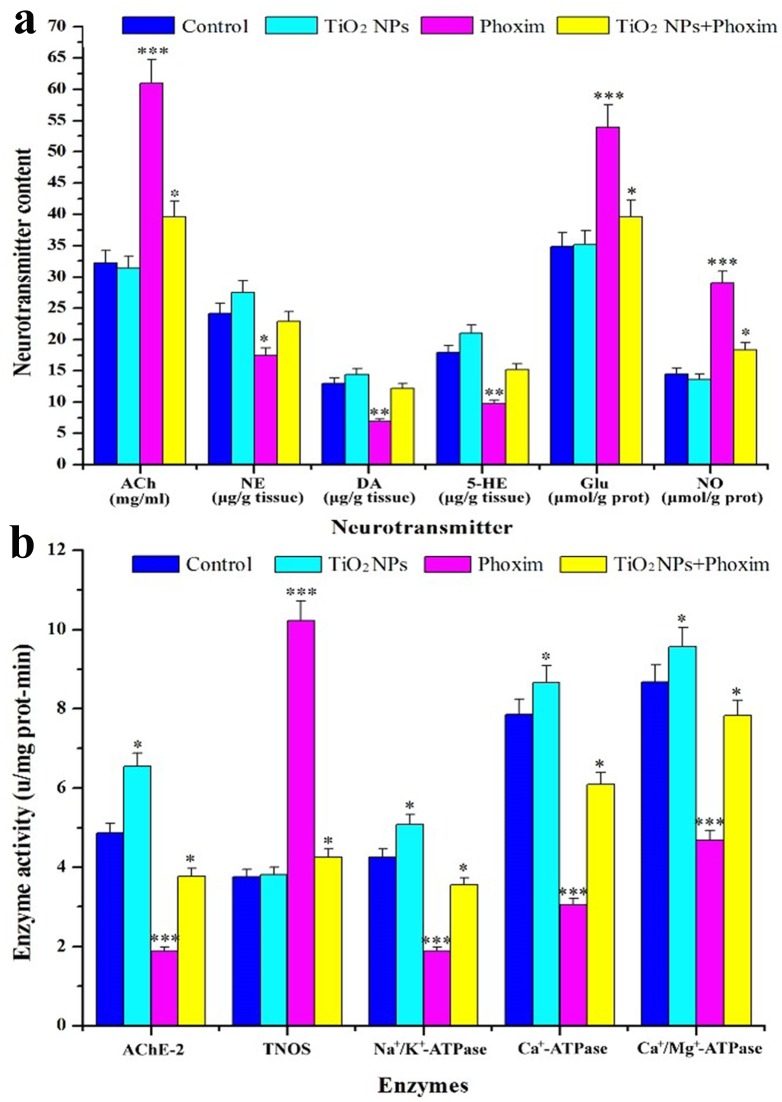
Effects of TiO_2_ NPs on nerve conduction in the brain of phoxim-exposed fifth-instar larvae. *p<0.05, **p<0.01, and ***p<0.001. Values represent means ± SEM (*N* = 5). (a) Neurotransmitter contents, (b) Enzyme activity.

The contents of neurotransmitters, including ACh, Glutamate (Glu), and nitric oxide (NO), in the brains of fifth-instar larvae in the phoxim-exposed group were higher than those of the control, while the contents of norepinephrine (NE), dopamine (DA), and 5-hydroxytryptamine (5-HT) were otherwise decreased significantly by phoxim exposure (Fig. 4a). Pretreatments with TiO_2_ NPs reversed the changes in the contents of NE, DA, 5-HT, ACh, Glu, and NO (Fig. 4a). We also observed that phoxim exposure significantly inhibited the activities of AChE, Na^+^/K^+^-ATPase, Ca^2+^-ATPase, and Ca^2+^/Mg^2+^-ATPase and promoted the activity of total nitric oxide synthase (TNOS) in the brain, while TiO_2_ NPs significantly promoted the activities of AChE, Na^+^/K^+^-ATPase, Ca^2+^-ATPase, Ca^2+^/Mg^2+^-ATPase, and AChE and inhibited the activity of TNOS (Fig. 4b). These results demonstrated that phoxim exposure altered the releases of neurotransmitters and the activities of several important enzymes in the nerve conduction in *B. mori* larvae brain, while TiO_2_ NPs were able to reverse such changes.

### Oxidative stress

**Figure 5:**
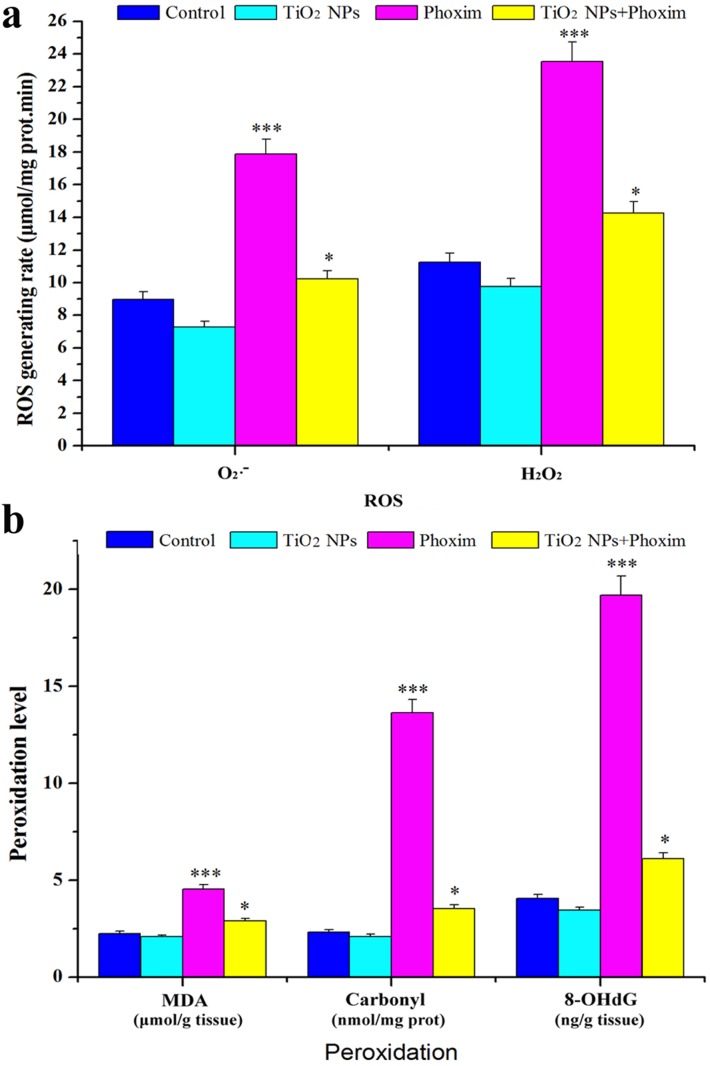
Effects of TiO_2_ NPs on oxidative stress in brain of phoxim-exposed fifth-instar larvae. *p<0.05, and ***p<0.001. Values represent means ± SEM (*N* = 5). (a) ROS production, (b) Levels of lipid, protein, and DNA peroxidation.

As shown in Figure 5a, phoxim exposure significantly promoted the production of ROS species, such as O_2_^−^ and H_2_O_2_, in larval brains (*P*<0.001) at 48 h, while TiO_2_ NPs attenuated such enhancement in ROS production (*P*<0.05). The ROS production was further demonstrated by the measurements of the levels of lipid peroxidation (MDA), protein peroxidation (protein carbonyl, PC), and DNA damage (8-hydroxy deoxyguanosine, 8-OHdg) in the larval brain (Fig. 5b). Significantly increased MDA, protein carbonyl, and 8-OHdG were observed in the phoxim-exposed midguts, but the increases became much lower with the combined treatments (Fig. 5b). It suggested that TiO_2_ NPs treatments decreased ROS accumulation, which may lead to attenuated peroxidation of lipids, proteins, and DNAs in the larval brains under phoxim-induced toxicity.

### Digital Gene Expression Profile

To investigate the molecular mechanisms of reduced nerve toxicity by TiO_2_ NPs under phoxim stress in the brain of *B. mori*, we adopted the DGE method to detect the differences in gene expression in the brain among the control-, TiO_2_ NPs-, phoxim-, and TiO_2_ NPs + phoxim-treated larvae at 48 h. Compared with those of the control group, 288, 295, and 472 genes were expressed significantly differently in the TiO_2_ NPs group (Fig. S1), the phoxim group (Fig. S2), and the TiO_2_ NPs + phoxim group (Fig. S3), respectively, with 117, 64, and 48 genes being up-regulated, respectively, and 171, 231, and 424 genes being down-regulated, respectively. The genes with differential expression were classified by Gene Ontology (GO) classification analysis into 12 groups, which were oxidative stress, stress response, metabolic process, cell component, transport, transcription-related, translation-related, growth and development, nerve conduction, immune response, cell cycle, and apoptosis (Tab. S1, S2, S3).

### Gene Expression Detection by qRT-PCR

Combine with DGE assay, histopathological and ultrastructure evaluation, we hypothesized TiO_2_ NPs pretreatment might decrease the expression changes of genes essential in maintain normal physiological activity. To validate the hypothesis, we performed qRT-PCR for several genes that are involved in neurotoxicity, ion transport, oxidative stress and apoptosis. In the present study, *actin3* was used as the internal reference gene. As shown in Table 1, the expression level of *ace1* was significantly increased by 19.45-fold after 48 h of phoxim exposure, while the expression levels of *H^+^ transporting ATP synthase*, *vacuolar ATP synthase*, *SOD* and *TPx* were significantly reduced by 63%, 55%, 74% and 35% respectively. However, the expression changes of *ace1*, *H^+^ transporting ATP synthase* and *vacuolar ATP synthase* were 6.69-fold, 0.78-fold, 0.93-fold, 0.81-fold and 0.93-fold respectively for TiO_2_ NPs + phoxim-treated brains. Moreover, the expression of *cytochrome-c* was up-regulated by 1.21-fold in the phoxim-exposed brain, but by 1.02-fold in the TiO_2_ NPs + phoxim treated group. All the qRT-PCR data were consistent with those of DGE assay (Tab. 1). In order to explore whether phoxim stress induced apoptosis through the mitochondria/cytochrome-c pathway, the expression of four additional genes regulating mitochondria apoptosis pathway were determined by qRT-PCR. As shown in Table 1, compared with the control group, the expression levels of three pro-apoptotic genes, *Bm109*, *caspase-9*, and *caspase-3* were changed by 3.0-fold, 2.51-fold, and 3.07-fold, respectively in the phoxim-exposed group, and by 2.42-fold, 2.2-fold, and 2.45-fold, respectively in the TiO_2_ NPs + phoxim-treated group. On the other hand, the mRNA levels of *BmIAP*, an apoptosis inhibitor gene, were down-regulated by 0.785-fold under phoxim stress, but by 0.91-fold in the TiO_2_ NPs + phoxim-treated group, respectively. These results indicated that TiO_2_ NPs treatment decreased expression alterations of these genes involved in neurotoxicity, ion transport, oxidative stress and apoptosis in the brain under phoxim stress.

**Table 1:** Comparison between fold-difference with qRT-PCR results and DGE assay in each group.

Gene	TiO_2_ NPs/Control		Phoxim/Control		TiO_2_ NPs + Phoxim/Control	
	qRT-PCR(Fold)	DGE(log_2_ value)	qRT-PCR(Fold)	DGE(log_2_ value)	qRT-PCR(Fold)	DGE(log_2_ value)
*ace1*	0.823	0.068	19.453***	0.956	6.689***	0.583
*H+ tATPase*	2.143	1.324	0.373***	−0.303	0.779**	−0.148
*vATPase*	1.065	0.547	0.453**	−1.236	0.933	−1.061
*SOD*	1.317	0.218	0.263**	−0.627	0.808	−0.472
*TPx*	1.204	0.484	0.649*	−0.680	0.930	−0.406
*Bm109*	0.849	No difference	2.999**	No difference	2.416**	No difference
*BmIap*	1.026	No difference	0.785*	No difference	0.905	No difference
*caspase-9*	0.979	No difference	2.513**	No difference	2.204**	No difference
*caspase-3*	0.970	No difference	3.065***	No difference	2.451**	No difference
*cytochrome c*	0.953	−0.085	1.214*	0.294	1.021	0.099

*p<0.05, **p<0.01, and ***p<0.001.

Values represent means ± SEM (*n* = 5).

## Discussion

The insect brain, a part of CNS, is essential in regulating nerve conduction, growth and development. It has been reported that the brain of *B. mori* is the target organ of nerve agent phoxim. In the present study, the body weight and survival rate of *B. mori* were significantly reduced by phoxim (Fig. 1), and severe brain damage was observed (Fig. 2), while pretreatment with TiO_2_ NPs protected the brain (Fig. 2). In addition, TiO_2_ NPs decreased the severe apoptosis of brain cells after phoxim exposure (Fig. 3) and protected larvae from anomalous nerve conduction (Fig. 4) and excessive ROS production (Fig. 5). Furthermore, we adopted DGE assay and qRT-PCR method to explore the molecular mechanisms of reduced nerve toxicity by TiO_2_ NPs in the phoxim-exposed brain of *B. mori*, the main results were divided into three parts and discussed below.

### Nerve conduction

It has been reported that vacuolar-type ATPases (V-ATPases) produce proton-motives that are indispensable for ion transports and the energization of membrane transport in insect systems [19]. In the present study, the expression of *H^+^ transporting ATP synthase* and *vacuolar ATP synthase* was down-regulated under phoxim stress in the brain of *B. mori* larvae, which was reversed by TiO_2_ NPs. Furthermore, the activity of Na^+^/K^+^-ATPase that maintains the balance of K^+^ and Na^+^ concentrations in the organisms was inhibited in the brain under phoxim stress, which resulted in physiological damages and cellular homeostasis disturbance [20]; these changes could also be mitigated by TiO_2_ NPs pretreatments. Besides, Ca^2+^ concentration that is essential for ion transport and nerve conduction is regulated by Ca^2+^-ATPase and Ca^2+^/Mg^2+^-ATPase in eukaryotic cells, and defects in these enzymes seriously compromise the normal functions of cells [21]. Similar to the finding of inhibited activity of Ca^2+^/Mg^2+^-ATPase by pyrethroids [22], we observed inhibited activities of Ca^2+^-ATPase and Ca^2+^/Mg^2+^-ATPase by phoxim in the brain of *B. mori* in our study. However, the activities of the two enzymes were only slightly inhibited in the TiO_2_ NPs + phoxim group. These changes in gene expression and enzymatic activity in the brain are expected to lead to abnormal concentrations of neurotransmitters that are important in nerve conduction. Monoamine neurotransmitters, such as 5-HT, DA, and NE, are closely related to learning, memory, and normal behaviors [14], [23]. In this study, the contents of 5-HT, DA, and NE were decreased significantly by phoxim exposure, while those in the TiO_2_ NPs + phoxim-treated larvae were similar to the control. The contents of several amino acid neurotransmitters, such as ACh, Glu, and NO, were increased significantly by phoxim exposure.

It was reported that inhibition of the amino acid neurotransmitter AChE is the main mechanism of OP pesticides [2]. AChE catalyzes the hydrolysis of the excitatory neurotransmitter ACh into choline and acetic acid, which terminates nerve impulses on postsynaptic membrane [24]. Therefore, inhibited AChE activity results in increased ACh contents and continuing nerve impulses. In the current study, a significant inhibition of AChE activity was observed in the brain of phoxim-exposed larvae (Fig. 4b). However, the expression of *ace1* was actually up-regulated, likely a compensation for the inhibited AChE activity. This is consistent with the finding in a previous study [1]. TiO_2_ NPs treatments mitigated the inhibition of AChE activity (Fig. 4b) and down-regulated the *ace1* expression.

Glu, another excitatory amino acid neurotransmitter, binds to NMDA receptors to promote the influx of extracellular Ca^2+^ [25], [26] and enhance the activity of calcium-dependent proteases, such as TNOS that is involved in the release of NO. We observed that phoxim exposure significantly increased Glu contents, TNOS activity, and NO contents, while TiO_2_ NPs pretreatments reversed such increases. The free radical NO modulates neuronal functions by increasing the release of neurotransmitters [27], and NO can be oxidized to peroxinitrite (ONOO-) that may cause neuronal damages and induce apoptosis [26]. Therefore, the reversed changes in Glu contents, TNOS activity, and NO contents may further explain the protective effects of TiO_2_ NPs against phoxim-induced damages in the brain of *B. mori*.

### Oxidative stress

Previous study had shown mitochondria may be the primary target of OP-initiated cytotoxicity [28]. They play crucial roles in oxidative stress [29], as the levels of ROS species, such as O_2_^−^ and H_2_O_2_, are related to the respiratory chain, substrate dehydrogenases in the matrix, monoamine oxidase, and cytochrome P450 [30]. When mitochondria are damaged, the ROS levels are usually increased significantly, causing oxidative damages to lipids, proteins, and DNA. These oxidative damages generate peroxidation products, such as MDA, PC, and 8-OHdG [31]–[33], which may induce apoptosis and necrosis. However, many stress response proteins are associated with the removal of ROS in insects, such as SOD that is the primary antioxidant enzyme catalyzing the dismutation of superoxide radicals to hydrogen peroxide and oxygen [34] and TPx that removes hydrogen peroxide and alkyl hydroperoxides [35]. In the present study, the expression of both *SOD* and *TPx* was significantly inhibited after 48 h of phoxim exposure, along with significantly increased contents of O_2_^•−^ and H_2_O_2_ (Fig. 5a), significantly improved levels of MDA, PC, and 8-OHdG (Fig. 5b), and swelling mitochondria and broken mitochondria crista (Fig. 3c). However, in the TiO_2_ NPs group and the TiO_2_ NPs + phoxim group, the morphology of cells and mitochondria were normal, indicating that TiO_2_ NPs protected the brain of *B. mori* from phoxim stress by regulating the expression of genes important in oxidative stress and mitochondria respiratory chain.

### Apoptosis

Many pesticides have been shown to cause apoptosis and necrosis [36]. The accumulation of ROS and peroxidation may also promote the release of mitochondrial cytochrome c [37]. Once released to the cytoplasm, cytochrome c binds to the apoptotic protease activating factor-1 (Apaf-1) and pro-caspase-9 to form a tripolymer protein complex. The tripolymers form apoptosomes that activate caspase-9, an initiator caspase in the mitochondria/cytochrome-c pathway, and caspase-3 [38]. In the present study, over-expression of *cytochrome-c*, *caspase-9* and *caspase-3* was observed in phoxim-exposed brains, while TiO_2_ NPs treatments mitigated the over-expression. Moreover, apoptosis is regulated by many apoptotic associated proteins, such as Bcl-2 and the inhibitor of apoptosis proteins (IAP) [39], [40]. In *B. mori*, Bm109 is homologous to the anti-apoptotic Bcl-2 family proteins within the four conserved BH regions. Bm109 has been reported to up-regulate apoptosis by participating in the translocation of Bax to mitochondria and the release of cytochrome c [41], [42]. In this study, TiO_2_ NPs mitigated the over-expression of *Bm109* and the down-expression of *BmIAP*, a specific inhibitor of caspase-9 [43], under phoxim stress in the brain of *B. mori*. We also observed cell debris, swelled mitochondria, and broken mitochondria crista by histological and ultrastructure photomicrographs (Figs. 2c, 3c), indicating that phoxim induces apoptosis through the mitochondria/cytochrome-c pathway, and that TiO_2_ NPs treatments can mitigate mitochondrial damages and block apoptosis in the brain of *B. mori* under phoxim stress.

## Conclusion

The results from this study indicate that TiO_2_ NPs can reduce the phoxim-induced changes in the expression of genes and the activity of enzymes that regulate nerve conduction, oxidative stress and apoptosis, and relieve phoxim-induced physiological disorders and brain damages in *B. mori*. Our study may promote the application of TiO_2_ NPs in reducing pesticide toxicity in *B. mori* in the future, although further investigations are needed to reveal the specific mechanisms of the effects of TiO_2_ NPs on phoxim exposure.

## Materials and Methods

### Insects and Chemicals

The larvae of *B. mori* (*Bombyx mori L* Qiufeng × baiyu), which were maintained in our laboratory, were reared on mulberry leaves under 12-h light/12-h dark cycles for this study.

Nanoparticulate anatase TiO_2_ was prepared through controlled hydrolysis of titanium tetrabutoxide. Detailed synthesis and characterization of TiO_2_ NPs have been described previously [44].

Phoxim was purchased from Sigma Co. at 98.1% purity.

### Preparation of phoxim and TiO_2_ NPs solutions

TiO_2_ NPs powder was dispersed onto the surface of 0.5% Hydroxypropylmethylcellulose (HPMC) (w/v), suspended, sonicated for 30 min, and mechanically vibrated for 5 min. Phoxim was dissolved in acetone to prepare the stock solution, which was diluted with water into different concentrations for analysis. 0.5% HPMC was used as the suspending agent.

### Resistance measurement

In the pre-experiment, we tried 1, 2, 5, 10, and 15 mg/L TiO_2_ NPs suspensions in fifth-instar larvae and determined that the optimum concentration for larvae growth was 5 mg/L for further experiments. The Lethal Concentration 50 (*LC*_50_) of phomix in *B. mori* was 7.86 µg/mL, and 4 µg/mL was used as the concentration in further experiments. 100 g of fresh mulberry, *Morus albus (L.)*, leaves were dipped in 5 mg/L TiO_2_ NPs suspension for 1 min, followed by dipping in the solution of 4 µg/mL phoxim for 1 min.

After being air-dried, TiO_2_ NPs-treated leaves were used to feed *B. mori* instar larvae three times a day until the second day of fifth-instar larvae. Fresh leaves treated with 0.5% HPMC served as controls. Later, phoxim-treated leaves were used to feed *B. mori* larvae three times a day from the third day, before the silkworms (1,000 in each group) were fed with either TiO_2_ NPs-treated leaves or the control fresh leaves under long-day photoperiods (16 h light: 8 h dark) at 25°C and about 75% relative humidity. Each experiment was repeated three times with 200 larvae. The mortality of larvae was counted 48 h later.

To measure the resistance of larvae, the body weight and survival rate of larvae was counted 48 h later by the method of our previous study [4] with minor modifications.

### Brain tissue collection

Forty eight hours after phoxim treatments, 100 fifth-instar larvae were selected randomly from each group. Larval brains were collected and frozen at −80°C for subsequent antioxidant assay.

### Histopathological evaluation of brain

All histopathological examinations were performed using standard laboratory procedures. Brains of five larvae of each group were embedded in paraffin, sliced (5 µm thickness), placed onto glass slides, and stained with hematoxylin–eosin (HE). Stained sections were evaluated by a histopathologist who was unaware of the treatments using an optical microscope (Nikon U-III Multi-point Sensor System, Japan).

### Observation of brain ultrastructure

Brains of five larvae of each group were fixed in freshly prepared 0.1 M sodium cacodylate buffer with 2.5% glutaraldehyde and 2% formaldehyde, before being treated at 4°C with 1% osmium tetroxide in 50 mM sodium cacodylate (pH 7.2–7.4) for 2 h. Staining was performed overnight with 0.5% aqueous uranyl acetate. After serial dehydration with ethanol (75, 85, 95, and 100%), the specimens were embedded in Epon 812 and sliced. Ultrathin sections were treated with uranyl acetate and lead citrate and observed with a HITACHI H600 TEM (HITACHI Co., Japan). The apoptosis in brain was determined by observing the changes in nuclear morphology (e.g., chromatin condensation and fragmentation).

### Oxidative stress assay of brain

ROS (O_2_^−^ and H_2_O_2_) production, MDA levels, protein carbonyl (PC), and 8-OHdG in brain tissues were assayed using commercial enzyme-linked immunosorbent assay (ELISA) kits (Nanjing Jiancheng Bioengineering Institute, Jiangsu, China) following the manufacturer’s instructions.

### Assay of enzymatic activities

For enzymatic activity determinations, brain tissues were homogenized in 10 volumes of 0.15 M NaCl. A quantity of homogenate was used to the activies of different enzymes. The activities of AChE, Ca^2+^-ATPase, Ca^2+^/Mg^2+^-ATPase, Na^+^/K^+^-ATPase, and TNOS in the brain were spectrophotometrically measured with commercial kits (Nanjing Jiancheng Bioengineering Institute, China) targeting the oxidation of oxyhaemoglobin to methaemoglobin by nitric oxide.

### Determination of neurochemicals

The homogenate of brains was centrifuged at 12,000 g for 20 min at 4°C. The concentrations of DA, 5-HT, NE, and ACh were spectrophotometrically measured with commercially kits (Nanjing Jiancheng Bioengineering Institute, China).

Glu concentrations were measured using commercial kits (Nanjing Jianchen Biological Institute, China), and the standard curves were produced by using standard Glu stock solutions. Glu levels in the samples were detected using a spectrophotometer at 340 nm and expressed as µmol/g prot. The concentration of NO in the brain was measured using a commercial kit (Nanjing Jiancheng Bioengineering Institute, China). The OD value was determined by a spectrophotometer (U-3010, Hitachi, Japan). Results of NO were read with OD value at 550 nm. The results were calculated using the following formula: NO (µmol/L) = (Asample−Ablank)/(Astandard- Ablank)×20(µmol/L).

### Total RNA isolation

Total RNA was extracted from brain samples using the Trizol reagent (Takara, Japan) and treated with DNase to remove potential genomic DNA contamination. The quality of RNA was assessed by formaldehyde agarose gel electrophoresis and was quantitated spectrophotometrically.

### DGE library preparation, sequencing, tag mapping and evaluation of DGE libraries

For RNA library construction and deep sequencing, equal quantities of brain RNA samples (n = 3) were pooled for the control group and the treated group, respectively. Approximately 6 µg of RNA representing each group were submitted to Solexa (now Illumina Inc.) for sequencing. The detailed methodology were performed in our previous study [45].

### qRT-PCR analysis

The specific primers for the 11 genes are listed in Table S4. The internal reference gene was *actin3*. qRT-PCR was performed using the 7500 Real-time PCR System (ABI) with SYBR Premix Ex *Taq*™ (Takara, Japan) according to the manufacturer’s instructions. The qRT-PCR analysis was carried out following the method described in previous studies [1], [5].

### Statistical Analysis

Statistical analyses were performed using the SPSS 19 software. Data are expressed as means ± standard error of the mean (SEM). One-way analysis of variance (ANOVA) was carried out to compare the differences of means of the multigroup data. Dunnett’s test was performed when each dataset was compared with the solvent-control data. Statistical significance for all tests was judged at a probability level of 0.05 (*P*<0.05).

## Supporting Information

Figure S1 Functional categorization of 295 genes which significantly altered by phoxim exposure. Genes were functionally classified based on the ontology-driven clustering approach of PANTHER.
(DOC)

Figure S2 Functional categorization of 288 genes which altered by TiO_2_ NPs pretreatment. Genes were functionally classified based on the ontology-driven clustering approach of PANTHER.
(DOC)

Figure S3 Functional categorization of 472 genes which altered by TiO_2_ NPs + phoxim exposure. Genes were functionally classified based on the ontology-driven clustering approach of PANTHER.
(DOC)

Table S1 Genes related to oxidative stress, stress response, metabolic process, cell component, transport, transcription, translation, growth and development, signal transduction, immune response, cell cycle and apoptosis altered significantly by phoxim exposure.
(DOC)

Table S2 Genes related to oxidative stress, stress response, metabolic process, cell component, transport, transcription, translation, growth and development, signal transduction, immune response, cell cycle and apoptosis altered significantly by TiO_2_ NPs exposure.
(DOC)

Table S3 Genes related to oxidative stress, stress response, metabolic process, cell component, transport, transcription, translation, growth and development, signal transduction, immune response, cell cycle and apoptosis altered significantly by TiO_2_ NPs + phoxim exposure.
(DOC)

Table S4 Primer pairs for qRT-PCR in the gene expression analysis.
(DOC)
