# Supplementary material for: Molecular Mechanisms of Reduced Nerve Toxicity by Titanium Dioxide Nanoparticles in the Phoxim-Exposed Brain of Bombyx mori
Source: PLoS One. 2014 Jun 27;9(6):e101062. doi: 10.1371/journal.pone.0101062 (PMC4074129; doi:10.1371/journal.pone.0101062)
Supplement: Figure S1 — Functional categorization of 295 genes which significantly altered by phoxim exposure. Genes were functionally classified based on the ontology-driven clustering approach of PANTHER. (DOC) [file pone.0101062.s001.doc]

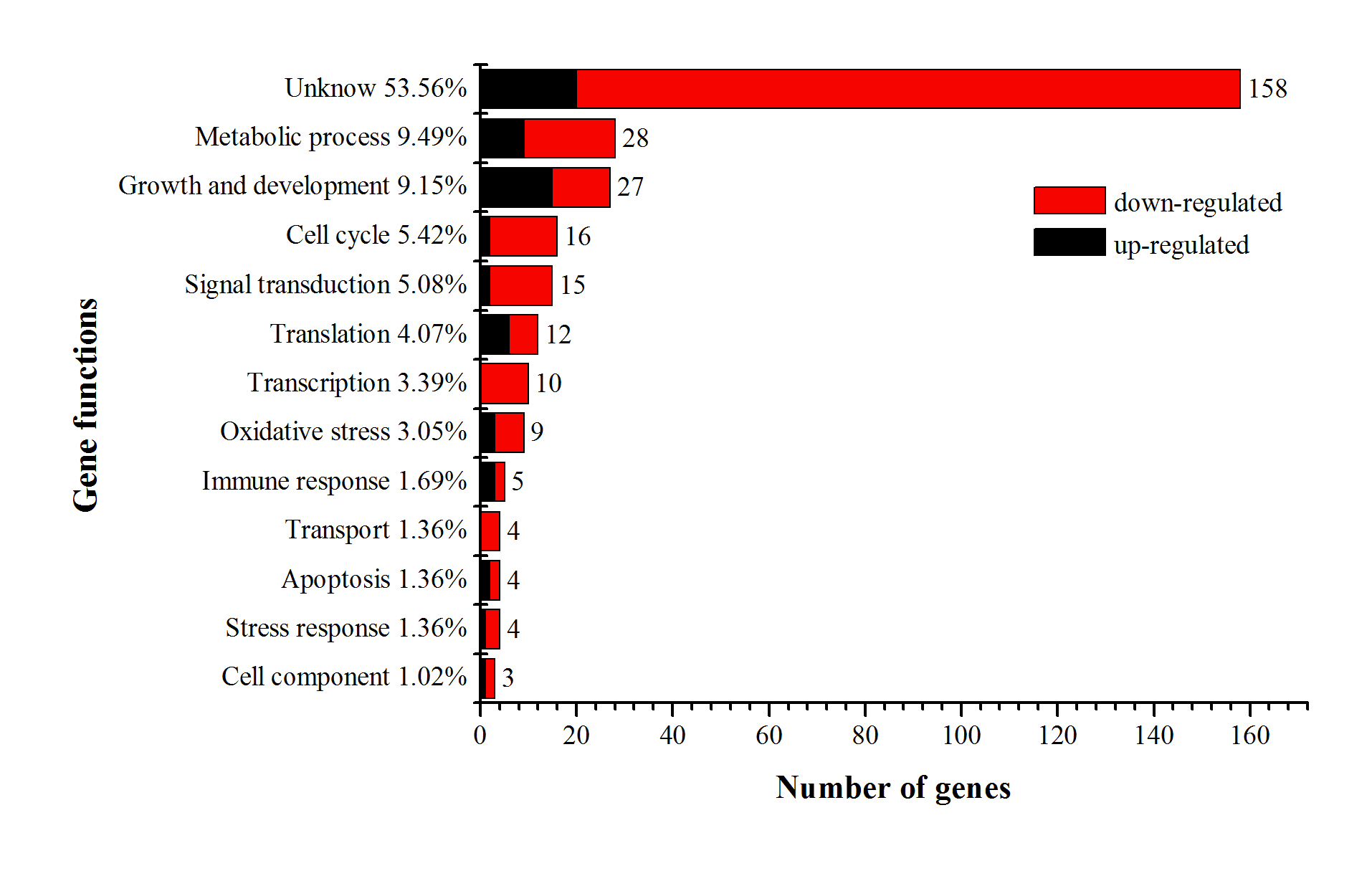


**Figure S1** Functional categorization of 295 genes which significantly altered by phoxim exposure. Genes were functionally classified based on the ontology-driven clustering approach of PANTHER.
