# Supplementary material for: Molecular Mechanisms of Reduced Nerve Toxicity by Titanium Dioxide Nanoparticles in the Phoxim-Exposed Brain of Bombyx mori
Source: PLoS One. 2014 Jun 27;9(6):e101062. doi: 10.1371/journal.pone.0101062 (PMC4074129; doi:10.1371/journal.pone.0101062)
Supplement: Figure S3 — Functional categorization of 472 genes which altered by TiO2 NPs + phoxim exposure. Genes were functionally classified based on the ontology-driven clustering approach of PANTHER. (DOC) [file pone.0101062.s003.doc]

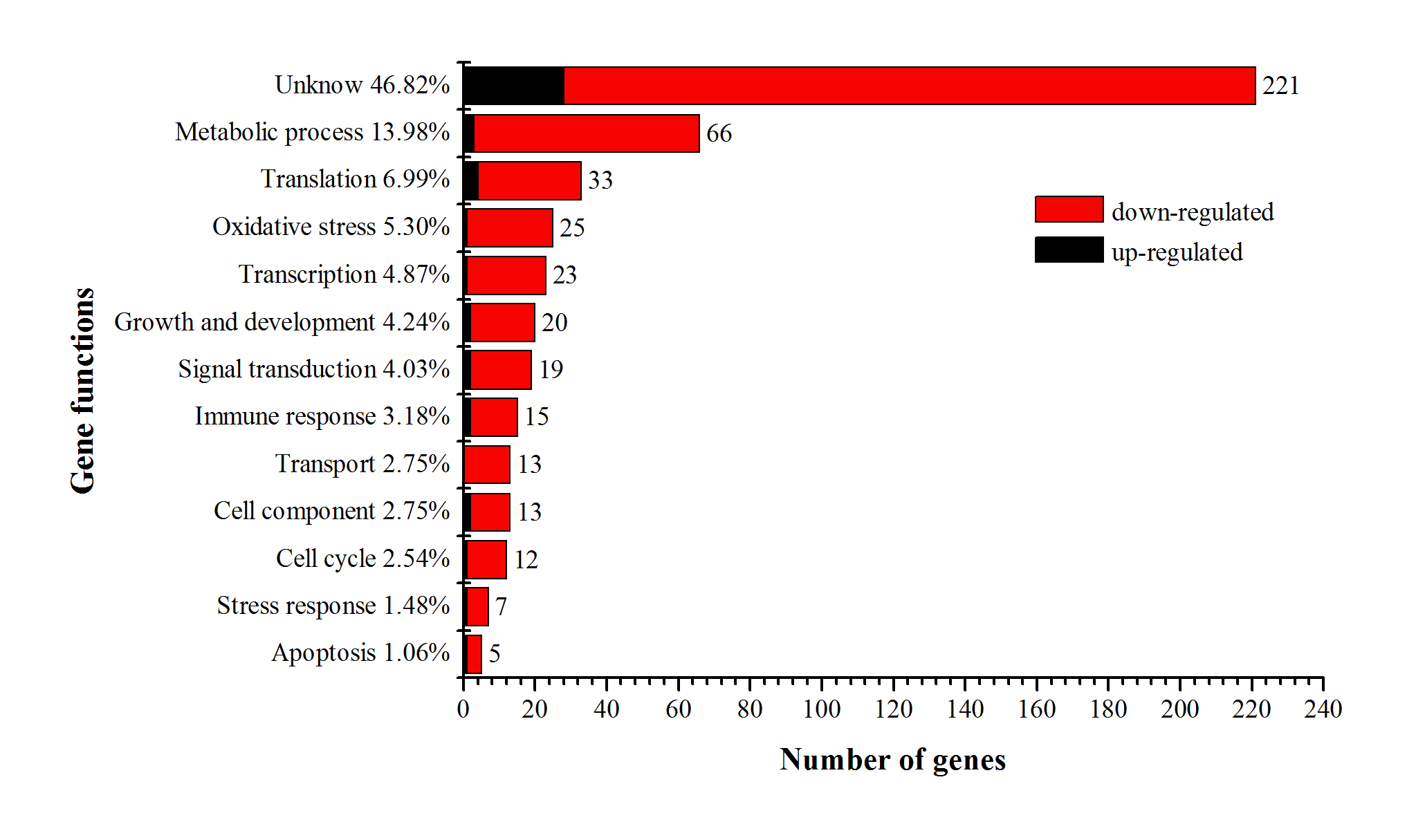


**Figure S3** Functional categorization of 472 genes which altered by TiO_2_ NPs+phoxim exposure. Genes were functionally classified based on the ontology-driven clustering approach of PANTHER.
