# Supplementary material for: Molecular Mechanisms of Reduced Nerve Toxicity by Titanium Dioxide Nanoparticles in the Phoxim-Exposed Brain of Bombyx mori
Source: PLoS One. 2014 Jun 27;9(6):e101062. doi: 10.1371/journal.pone.0101062 (PMC4074129; doi:10.1371/journal.pone.0101062)
Supplement: Table S1 — Genes related to oxidative stress, stress response, metabolic process, cell component, transport, transcription, translation, growth and development, signal transduction, immune response, cell cycle and apoptosis altered significantly by phoxim exposure. (DOC) [file pone.0101062.s004.doc]

**Table S1** Genes related to oxidative stress, stress response, metabolic process, cell component, transport, transcription, translation, growth and development, signal transduction, immune response, cell cycle and apoptosis altered significantly by phoxim exposure.

| **Oxidative stress** | | | | | | | | | | | | | |
| --- | --- | --- | --- | --- | --- | --- | --- | --- | --- | --- | --- | --- | --- |
| **Name** | **Sequence ID** | **P-Value** | **log2** | **Ontology** | **Name** | | **Sequence ID** | **P-Value** | | **log2** | | | **Ontology** |
| cytochrome P450 | BAD99563.1 | 4.52E-22 | 4.22757266 | oxidative stress | vesicle amine transport protein | | NP_001093281.1 | 7.56E-06 | | -1.182923588 | | | oxidoreductase activity |
| mitochondrial aldehyde dehydrogenase | EHJ66210.1 | 2.18E-07 | 2.602123052 | catalytic activity | hypothetical protein KGM_16514 | | EHJ70247.1 | 2.82E-16 | | -1.38481 | | | oxidoreductase activity |
| troponin C | EHJ76074.1 | 5.37E-11 | 1.622005843 | oxidoreductase activity | putative steroid dehydrogenase | | EHJ79287.1 | 1.60E-15 | | -1.44631 | | | oxidoreductase activity |
| superoxide dismutase | NP_001037084.1 | 0.264622 | -0.627247646 | antioxidant activity | desaturase | | AAQ74260.1 | 4.88E-14 | | -1.48316 | | | oxidoreductase activity |
| thiol peroxiredoxin | NP_001037083.1 | 6.72E-28 | -0.680111818 | oxidoreductase activity |  | |  |  | |  | | |  |
| **Stress response** | | | | | | | | | | | | | |
| **Name** | **Sequence ID** | **P-Value** | **log2** | **Ontology** | **Name** | | **Sequence ID** | **P-Value** | | **log2** | | | **Ontology** |
| heat shock protein 1 | NP_001091767.1 | 3.33E-160 | 1.706868301 | response to stress | proteasome subunit beta 7 | | NP_001040536.1 | 2.72E-28 | | -1.17162 | | | cellular response to stress |
| heat shock cognate protein | NP_001036892.1 | 2.14E-151 | -0.603228794 | response to stress | heat shock cognate 70 protein | | EHJ73638.1 | 3.48E-18 | | -1.63968 | | | response to stress |
| **Metabolic process** | | | | | | | | | | | | | |
| **Name** | **Sequence ID** | **P-Value** | **log2** | **Ontology** | **Name** | | **Sequence ID** | **P-Value** | | **log2** | | | **Ontology** |
| hypothetical protein KGM_11849 | EHJ78758.1 | 1.31E-08 | 3.624490865 | hydrogen transport, metabolic process | poly A binding protein | | NP_001091823.1 | 3.03E-07 | | -1.26926 | | | nucleic acid binding, catalytic activity |
| putative salivary/fat body serine carboxypeptidase | EHJ78980.1 | 5.60E-221 | 3.317385789 | peptidase activity | prophenoloxidase activating enzyme precursor | | NP_001036832.1 | 1.29E-06 | | -1.28861 | | | endopeptidase activity |
| aliphatic nitrilase | NP_001165388.1 | 2.32E-07 | 2.478710681 | hydrolase activity, | putative chromatin regulatory protein sir2 | | EHJ73249.1 | 1.36E-07 | | -1.36642 | | | binding |
| lysozyme precursor | NP_001037448.1 | 3.05E-05 | 2.129837438 | hydrolase activity, | asparagine synthetase | | NP_001037414.1 | 7.05E-19 | | -1.40409 | | | carbon-nitrogen ligase activity |
| serine proteinase-like protein precursor | NP_001040462.1 | 1.01E-05 | 1.455701947 | endopeptidase activity | hypothetical protein KGM_15891 | | EHJ66129.1 | 4.27E-06 | | -1.40644 | | | endonuclease activity, |
| cyclophilin-like protein | BAD90848.1 | 5.86E-10 | 1.375928462 | cis-trans isomerase activity | poly(A)-specific ribonuclease | | NP_001153677.1 | 6.06E-09 | | -1.40651 | | | 3'-5'-exoribonuclease activity |
| antitrypsin isoform 3 | ACT36278.1 | 3.73E-17 | 1.112087189 | endopeptidase inhibitor activity | hypothetical protein KGM_10825 | | EHJ65868.1 | 3.96E-07 | | -1.49768 | | | transaminase activity |
| hypothetical protein KGM_19988 | EHJ76797.1 | 4.80E-10 | 1.032400372 | phosphotransferase activity | dolichyl-phosphate mannosyltransferase | | NP_001040468.1 | 1.80E-23 | | -1.53587 | | | transferase activity |
| cationic peptide CP8 precursor | ABL76064.1 | 7.30E-17 | 1.014016053 | peptidase inhibitor activity | hypothetical protein KGM_02567 | | EHJ67837.1 | 2.31E-05 | | -1.55026 | | | nitrogen compound metabolic process |
| ubiquitin conjugating enzyme 4 | NP_001108475.1 | 1.50E-23 | -1.040101658 | ligase activity, forming carbon-nitrogen bonds | ubiquitin-conjugating enzyme E2M | | NP_001040241.1 | 6.61E-28 | | -1.55889 | | | small conjugating protein ligase activity |
| hypothetical protein KGM_07298 | EHJ68188.1 | 2.00E-10 | -1.062576569 | nucleoside-diphosphatase activity | hypothetical protein KGM_02043 | | EHJ67594.1 | 2.49E-08 | | -1.61311 | | | ligase activity, forming carbon-nitrogen bonds |
| cystathionine gamma-lyase | NP_001040113.1 | 7.04E-09 | -1.083477642 | carbon-sulfur lyase activity, cofactor binding | ornithine decarboxylase antizyme 1 | | NP_001037028.1 | 2.00E-14 | | -1.75931 | | | enzyme inhibitor activity |
| hypothetical protein KGM_08495 | EHJ78241.1 | 3.88E-14 | -1.189818431 | hydrolase activity | uridine phosphorylase | | EHJ70429.1 | 5.52E-07 | | -1.76458 | | | catalytic activity |
| putative slingshot dual specificity phosphatase | EHJ63863.1 | 7.84E-07 | -1.19734129 | phosphate metabolic process | hypothetical protein KGM_08817 | | EHJ66626.1 | 6.50E-05 | | -3.30965 | | | exopeptidase activity |
| **Cell component** | | | | | | | | | | | | | |
| **Name** | **Sequence ID** | **P-Value** | **log2** | **Ontology** | **Name** | | **Sequence ID** | **P-Value** | | **log2** | | | **Ontology** |
| muscle myosin heavy chain | BAG30740.1 | 4.12E-65 | 2.200252323 | muscle myosin complex, sarcomere | PIN2/TRF1-interacting protein | | EHJ63380.1 | 2.06E-07 | | -1.03996 | | | binding |
| hypothetical protein KGM_11118 | EHJ69096.1 | 1.24E-14 | -1.424275173 | microtubule cytoskeleton |  | |  |  | |  | | |  |
| **Transport** | | | | | | | | | | | | | |
| **Name** | **Sequence ID** | **P-Value** | **log2** | **Ontology** | **Name** | | **Sequence ID** | **P-Value** | | **log2** | | **Ontology** | |
| hypothetical protein KGM_12961 | EHJ72634.1 | 1.55E-07 | -1.960471636 | amine transmembrane transporter activity | globin 1 | | NP_001136083.1 | 4.80E-19 | | -1.57229 | | iron ion binding, gas transport | |
| putative sugar transporter | EHJ69094.1 | 5.13E-05 | -1.703606997 | sugar transporter | putative Aktip protein | | EHJ76935.1 | 5.67E-10 | | -1.01874 | | endosome transport | |
| **Transcription** | | | | | | | | | | | | | |
| **Name** | **Sequence ID** | **P-Value** | **log2** | **Ontology** | **Name** | | **Sequence ID** | **P-Value** | | **log2** | | **Ontology** | |
| putative importin alpha 1a | EHJ74319.1 | 7.88E-05 | -2.397876948 | carbon catabolite regulation of transcription | hypothetical protein KGM_14516 | | EHJ76983.1 | 5.12E-08 | | -1.56351 | | nucleic acid binding | |
| transcription initiation factor IIE subunit beta | EHJ64456.1 | 9.46E-19 | -1.724267704 | gene expression | hypothetical protein KGM_10426 | | EHJ63902.1 | 1.26E-08 | | -1.38235 | | transcription elongation | |
| cleavage and polyadenylation specific factor 4 | NP_001040511.1 | 5.00E-07 | -1.699689682 | transition metal ion binding | receptor guanylyl cyclase GC-II | | AAN16469.1 | 2.29E-11 | | -1.25472 | | cyclase activity,  lyase activity | |
| putative LIM homeobox 1b | EHJ79142.1 | 4.80E-05 | -1.661864139 | metal ion binding | hypothetical protein KGM_15551 | | EHJ79046.1 | 4.26E-21 | | -1.16708 | | sequence-specific  DNA binding RNA polymerase II transcription activity | |
| putative Forkhead box protein E1 | EHJ71148.1 | 8.78E-52 | -1.58816341 | DNA binding | putative histone acetyltransferase | | EHJ71970.1 | 1.51E-06 | | -1.08689 | | binding,RNA polymerase activity | |
| **Translation** | | | | | | | | | | | | | |
| **Name** | **Sequence ID** | **P-Value** | **log2** | **Ontology** | **Name** | | **Sequence ID** | **P-Value** | | **log2** | | **Ontology** | |
| ribosomal protein L32 | NP_001091752.1 | 3.43E-267 | 1.223892813 | structural molecule activity | putative aminoacyl-tRNA synthetase | | EHJ77748.1 | 5.25E-25 | | -1.01173 | | aminoacyl-tRNA ligase activity | |
| ribosomal protein L7A | NP_001037138.1 | 4.00E-304 | 1.22310417 | ribonucleoprotein complex biogenesis | PREDICTED: similar to arginyl-tRNA synthetase | | XP_966449.1 | 9.85E-06 | | -1.11241 | | ligase activity | |
| seryl-tRNA synthetase | AEB26319.1 | 3.09E-09 | 1.172916676 | aminoacyl-tRNA ligase activity | alanine--tRNA ligase, cytoplasmic | | NP_001037452.1 | 3.01E-09 | | -1.18356 | | aminoacyl-tRNA ligase activity | |
| eukaryotic translation initiation factor 3 subunit I | NP_001040433.1 | 1.24E-32 | 1.07252019 | translation factor activity, nucleic acid binding | hypothetical protein KGM_08001 | | EHJ69245.1 | 2.67E-07 | | -1.26128 | | aminoacyl-tRNA ligase activity | |
| ribosomal protein L37 | NP_001037247.1 | 4.18E-14 | 1.062313445 | RNA binding,structural molecule activity | putative tyrosyl-tRNA synthetase | | EHJ76165.1 | 1.00E-05 | | -1.38399 | | aminoacyl-tRNA ligase activity | |
| ribosomal protein S12 | NP_001037568.1 | 1.72E-62 | 1.0251736 | structural molecule activity | hypothetical protein AND_17160 | | EFR21355.1 | 6.09E-13 | | -1.60658 | | aminoacyl-tRNA ligase activity, | |
| **Growth and development** | | | | | | | | | | | | | |
| **Name** | **Sequence ID** | **P-Value** | **log2** | **Ontology** | **Name** | **Sequence ID** | | | **P-Value** | **log2** | | **Ontology** | |
| antennal esterase CXE14 | AEJ38205.1 | 2.63E-22 | 5.365072587 | antennal esterase | miniparamyosin | ACM17460.1 | | | 3.51E-15 | 1.072852 | | nucleoside-triphosphatase activity | |
| juvenile hormone binding protein brP-2095 precursor | NP_001036987.1 | 3.60E-211 | 5.134142341 | juvenile hormone associated | putative Dolichyl-phosphate beta-glucosyltransferase | EHJ67139.1 | | | 1.38E-11 | -1.00487 | | transferase activity, transferring glycosyl groups | |
| odorant binding protein LOC100301495 precursor | NP_001153663.1 | 1.20E-59 | 3.284758733 | odorant binding | putative E3 ubiquitin ligase | EHJ74431.1 | | | 4.19E-06 | -1.09027 | | cell development | |
| sex-specific storage-protein 1 precursor | NP_001106747.2 | 1.04E-32 | 2.941925399 | sex-specific protein | diuretic hormone 31 precursor | NP_001124379.1 | | | 7.63E-12 | -1.23881 | | hormone activity | |
| diapause associated protein 2 | AFC35301.1 | 1.21E-11 | 2.086770469 | diapause associated | chitin synthase A | NP_001245291.1 | | | 1.11E-05 | -1.2814 | | acetylglucosaminyltransferase activity | |
| fibroin P25 | BAB39500.1 | 2.84E-06 | 1.995674075 | biosynthesis of silk protein | putative zinc finger protein | EHJ64457.1 | | | 8.05E-07 | -1.3889 | | metal ion binding | |
| muscle LIM protein isoform 1 | NP_001103762.1 | 6.67E-142 | 1.892211886 | transition metal ion binding | elongation factor 1 gamma | NP_001036852.1 | | | 1.44E-11 | -1.47954 | | translation factor activity | |
| odorant binding protein | BAH36759.1 | 2.42E-140 | 1.770737468 | binding | hypothetical protein KGM_22205 | EHJ67660.1 | | | 5.82E-07 | -1.48032 | | transition metal ion binding | |
| troponin T transcript variant B | ACN86367.1 | 1.36E-31 | 1.586507142 | cytoskeletal protein binding, metal ion binding | putative kinesin-associated protein | EHJ67981.1 | | | 2.02E-08 | -1.48403 | | binding | |
| hemolymph juvenile hormone binding protein precursor | AAF19267.1 | 6.14E-05 | 1.42241703 | juvenile hormone associated | roundabout, isoform B | EHJ64790.1 | | | 1.30E-06 | -1.72009 | | organ development | |
| chemosensory protein 7 precursor | NP_001037068.1 | 8.79E-27 | 1.379208325 | organ development | putative zinc finger, CCCH-type with G patch domain protein | EHJ69184.1 | | | 1.03E-08 | -1.91406 | | metal ion binding | |
| hypothetical protein KGM_05708 | EHJ68237.1 | 1.52E-05 | 1.345410933 | cytoskeletal protein binding | odorant binding protein | BAH79159.1 | | | 2.32E-12 | -3.72561 | | odorant binding | |
| sex-specific storage-protein 2 precursor | NP_001037590.1 | 1.30E-268 | 1.171838039 | sex-specific protein | juvenile hormone binding protein | BAH97095.1 | | | 1.93E-12 | -4.05272 | | juvenile hormone associated | |
| putative ecdysone oxidase | EHJ63852.1 | 1.85E-15 | 1.081955596 | ecdysone associated |  |  | | |  |  | |  | |
| **Signal transduction** | | | | | | | | | | | | | |
| **Name** | **Sequence ID** | **P-Value** | **log2** | **Ontology** | **Name** | | **Sequence ID** | **P-Value** | | **log2** | | **Ontology** | |
| H+ transporting ATP synthase O subunit isoform 1 | NP_001040526.1 | 9.15E-79 | 1.023064126 | hydrogen ion transmembrane transporter activity | putative serotonin receptor | | ABI33826.1 | 0.00011048 | | -1.442850157 | | serotonin receptor activity | |
| acetylcholinesterase type 1 | ABY50088.1 | 4.21E-06 | 0.955583473 | acetylcholine catabolic process | putative signal recognition particle 54 kDa protein | | NP_001091776.1 | 4.93E-11 | | -1.50916 | | peptide binding, pyrophosphatase activity | |
| H+ transporting ATP synthase gamma subunit | NP_001040428.1 | 0.025002 | -0.303181972 | hydrogen ion transmembrane transporter activity | importin-7 | | EHJ74083.1 | 5.94E-10 | | -1.60387 | | protein binding,protein transporter activity | |
| putative tubulin folding cofactor E | EHJ77807.1 | 2.38E-05 | -1.114727181 | synaptic transmission | putative sodium pump alpha subunit | | EHJ73788.1 | 4.68E-16 | | -1.70887 | | ATPase activity | |
| vacuolar ATP synthase subunit E | NP_001040451.1 | 1.28E-10 | -1.235683918 | hydrogen ion transmembrane transporter activity | CHH-like protein precursor | | NP_001106139.1 | 1.40E-07 | | -1.76137 | | hormone activity | |
| putative NMDA-type glutamate receptor 1 | EHJ78211.1 | 1.21E-07 | -1.315180537 | glutamate receptor activity | cation efflux protein/zinc transporter | | EHJ67100.1 | 2.34E-06 | | -1.86206 | | cation transport | |
| putative Insulin receptor precursor | EHJ69301.1 | 8.01E-11 | -1.352666641 | signal transducer activity | vacuolar ATP synthase catalytic subunit A | | NP_001091829.1 | 1.72E-05 | | -3.0261 | | hydrogen ion transmembrane transporter activity | |
| endoplasmic reticulum-resident kdel protein | XP_001655120.1 | 8.37E-10 | -1.370620972 | UDP-glucosyltransferase activity |  | |  |  | |  | |  | |
| **Immune response** | | | | | | | | | | | | | |
| **Name** | **Sequence ID** | **P-Value** | **log2** | **Ontology** | **Name** | | **Sequence ID** | **P-Value** | | **log2** | **Ontology** | | |
| fungal protease inhibitor F precursor | NP_001037532.1 | 2.26E-40 | 3.242349236 | fungal protease inhibitor | serine protease inhibitor 13 precursor | | NP_001139705.1 | 5.82E-06 | | -2.05029 | endopeptidase inhibitor activity, protein binding | | |
| 30kDa protein | ADQ89805.1 | 1.04E-103 | 2.048878123 | immune response | glutathione S-transferase sigma 1 | | NP_001037077.1 | 1.90E-16 | | -2.8265 | transferase activity | | |
| selenophosphate synthetase 1 | NP_001037388.1 | 1.07E-12 | 1.11042399 | kinase activity |  | |  |  | |  |  | | |
| **Cell cycle** | | | | | | | | | | | | | |
| **Name** | **Sequence ID** | **P-Value** | **log2** | **Ontology** | **Name** | | **Sequence ID** | **P-Value** | | **log2** | | **Ontology** | |
| cdc2-related kinase | NP_001037345.1 | 6.52E-07 | 1.359690448 | protein serine/threonine kinase activity | meiotic recombination 11 | | NP_001036845.1 | 9.04E-11 | | -1.33165 | | transition metal ion binding | |
| chaperonin subunit 6a zeta | NP_001040108.1 | 1.98E-23 | 1.035988503 | ATPase activity,protein binding | hypothetical protein KGM_02129 | | EHJ63237.1 | 7.32E-36 | | -1.39027 | | mitotic cell cycle | |
| PREDICTED: nucleolar GTP-binding protein 1 | XP_002935223.1 | 8.23E-142 | -1.029933612 | nucleoside-triphosphatase activity | small nuclear ribonucleoprotein G | | EHJ71789.1 | 9.86E-28 | | -1.45618 | | translation repressor activity, mRNA binding | |
| hypothetical protein KGM_05790 | EHJ68863.1 | 6.57E-06 | -1.080304797 | cell migration | hypothetical protein KGM_18080 | | EHJ70264.1 | 5.56E-08 | | -1.5809 | | G2/M transition DNA damage checkpoint | |
| putative protein arginine N-methyltransferase | EHJ76958.1 | 8.49E-15 | -1.084752498 | binding | hypothetical protein KGM_14990 | | EHJ66282.1 | 5.87E-15 | | -1.59796 | | binding | |
| male-specific lethal 3 | NP_001093308.1 | 3.05E-05 | -1.153424768 | binding | rothmund-thomson syndrome DNA helicase recq4 | | EHJ75407.1 | 3.57E-08 | | -1.75809 | | double-stranded DNA binding | |
| DNA-directed DNA polymerase epsilon 2 | EHJ64679.1 | 4.71E-10 | -1.170628515 | transferase activity | putative structural maintenance of chromosome | | EHJ68833.1 | 1.21E-19 | | -1.76988 | | binding | |
| PREDICTED: probable DNA mismatch repair protein Msh6-like | XP_003699487.1 | 6.04E-09 | -1.271947917 | binding | hypothetical protein KGM_07972 | | EHJ70338.1 | 5.64E-07 | | -2.12839 | | ATPase activity, nucleotide binding, tubulin binding | |
| **Apoptosis** | | | | | | | | | | | | | |
| **Name** | **Sequence ID** | **P-Value** | **log2** | **Ontology** | **Name** | | **Sequence ID** | **P-Value** | | **log2** | | **Ontology** | |
| putative mitochondrial NADH: ubiquinone oxidoreductase ESSS subunit | EHJ72986.1 | 2.21E-16 | 1.923954976 | mitochondrial NADH | hypothetical protein KGM_13696 | | EHJ66180.1 | 3.85E-52 | | -1.607444501 | | respiratory electron transport chain | |
| mitochondrial cytochrome c | ACF41193.1 | 0.757996 | 0.29376159 | electron carrier activity | DEAD box polypeptide 5 isoform 1 | | NP_001037582.1 | 1.41E-22 | | -1.26085 | | ATPase activity, coupled | |
