# Supplementary material for: Molecular Mechanisms of Reduced Nerve Toxicity by Titanium Dioxide Nanoparticles in the Phoxim-Exposed Brain of Bombyx mori
Source: PLoS One. 2014 Jun 27;9(6):e101062. doi: 10.1371/journal.pone.0101062 (PMC4074129; doi:10.1371/journal.pone.0101062)
Supplement: Table S2 — Genes related to oxidative stress, stress response, metabolic process, cell component, transport, transcription, translation, growth and development, signal transduction, immune response, cell cycle and apoptosis altered significantly by TiO2 NPs exposure. (DOC) [file pone.0101062.s005.doc]

**Table S2** Genes related to oxidative stress, stress response, metabolic process, cell component, transport, transcription, translation, growth and development, signal transduction, immune response, cell cycle and apoptosis altered significantly by TiO_2_ NPs exposure.

| **Oxidative stress** | | | | | | | | | |
| --- | --- | --- | --- | --- | --- | --- | --- | --- | --- |
| **Name** | **Sequence ID** | **P-Value** | **log2** | **Ontology** | **Name** | **Sequence ID** | **P-Value** | **log2** | **Ontology** |
| hypothetical protein KGM_11849 | EHJ78758.1 | 9.18E-31 | 5.270351 | oxidoreductase activity | hypothetical protein KGM_21119 | EHJ69997.1 | 0.000123 | -1.54939 | oxidoreductase activity |
| hypothetical protein TcasGA2_TC002989 | EFA00167.1 | 8.09E-08 | 1.510281 | hydrolase activity | cytochrome P450 9a20 | NP_001077079.1 | 3.36E-17 | -1.55837 | oxidoreductase activity |
| thioredoxin-like protein | NP_001091804.1 | 1.46E-13 | 1.280844 | disulfide oxidoreductase activity | 2-oxoglutarate dehydrogenase | EHJ69053.1 | 6.39E-05 | -1.68989 | oxidoreductase activity |
| lethal(3)neo18 | NP_001124377.1 | 1.36E-09 | 1.006633 | response to oxidative stress | cytochrome P450 333B11 | EHJ70454.1 | 7.90E-10 | -1.69841 | oxidative stress |
| thiol peroxiredoxin | NP_001037083.1 | 8.42E-22 | 0.484458 | oxidoreductase activity | short-chain dehydrogenease/reductase-like | NP_001040155.1 | 7.39E-06 | -1.90414 | catalytic activity |
| superoxide dismutase | NP_001037084.1 | 0.640556 | 0.218281 | antioxidant activity | lactate dehydrogenase | NP_001095933.1 | 1.61E-06 | -2.73943 | lactate dehydrogenase activity |
| desaturase | AAQ74260.1 | 5.31E-11 | -1.231 | oxidoreductase activity | vesicle amine transport protein | NP_001093281.1 | 8.34E-24 | -4.89592 | oxidoreductase activity |
| **Stress response** | | | | | | | | | |
| **Name** | **Sequence ID** | **P-Value** | **log2** | **Ontology** | **Name** | **Sequence ID** | **P-Value** | **log2** | **Ontology** |
| heat shock protein hsp23.7 precursor | NP_001036942.1 | 2.37E-20 | 2.677343 | response to stress | putative hsp70 binding protein | EHJ79248.1 | 3.65E-39 | 1.323384 | response to stress |
| heat shock protein 25.4 precursor | NP_001112375.1 | 2.44E-29 | 2.549269 | response to stress | heat shock cognate protein | NP_001036892.1 | 0 | 1.275306 | response to stress |
| DnaJ (Hsp40) homolog 4 | NP_001157382.1 | 0.000109 | 2.118888 | response to stress | 33.6 kDa small heat shock protein | EHJ77259.1 | 1.39E-76 | 1.003488 | response to stress |
| heat shock protein hsp 19.9 | NP_001036984.1 | 4.22E-231 | 2.028807 | response to stress | DnaJ (Hsp40) homolog 3 | NP_001157381.1 | 9.26E-09 | -1.10222 | protein binding |
| 19.5 kDa heat shock protein | NP_001164470.1 | 4.54E-05 | 1.854249 | response to stress | DnaJ-26, partial | AFC01240.1 | 6.11E-06 | -1.71486 | response to stress |
| 19.8 kDa small heat shock protein | EHJ68318.1 | 0 | 1.558926 | response to stress | heat shock protein 1 | NP_001091767.1 | 5.85E-53 | -1.77692 | response to stress |
| proteasome subunit beta 7 | NP_001040536.1 | 1.20E-87 | 1.350893 | endopeptidase activity |  |  |  |  |  |
| **Metabolic process** | | | | | | | | | |
| **Name** | **Sequence ID** | **P-Value** | **log2** | **Ontology** | **Name** | **Sequence ID** | **P-Value** | **log2** | **Ontology** |
| lysozyme precursor | NP_001037448.1 | 6.10E-05 | 2.067923 | hydrolase activity | ferritin precursor | NP_001037584.1 | 0 | 1.008049 | iron ion binding |
| hypothetical protein KGM_20384 | EHJ75229.1 | 4.85E-11 | 1.562828 | nucleoside-triphosphatase activity | putative carbamoyl-phosphate synthase large chain | EHJ76789.1 | 1.59E-07 | -1.00375 | carboxyl- or carbamoyltransferase activity |
| hypothetical protein TcasGA2_TC002989 | EFA00167.1 | 8.09E-08 | 1.510281 | hydrolase activity | ubiquitin conjugating enzyme 4 | NP_001108475.1 | 3.50E-24 | -1.05338 | ligase activity, forming carbon-nitrogen bonds |
| coiled-coil-helix-coiled-coil-helix domain-containing protein 2, mitochondrial-like | NP_001093285.1 | 7.28E-209 | 1.428028 | hydrolase activity | DNA polymerase delta catalytic subunit | EHJ68324.1 | 3.27E-05 | -1.07893 | nucleic acid binding |
| poly(A)-specific ribonuclease | NP_001153677.1 | 3.00E-18 | 1.303672 | 3'-5'-exoribonuclease activity | ornithine decarboxylase antizyme 1 | NP_001037028.1 | 6.48E-08 | -1.10449 | enzyme inhibitor activity |
| bombyxin B-4 precursor | NP_001121792.1 | 3.68E-08 | 1.298074 | receptor binding | peptidylprolyl isomerase B precursor | NP_001040479.1 | 1.74E-05 | -1.10977 | cis-trans isomerase activity |
| putative Sirtuin 5 | EHJ71073.1 | 2.98E-12 | 1.294946 | transferase activity | hypothetical protein KGM_09610 | EHJ64233.1 | 4.39E-06 | -1.11895 | coenzyme binding,catalytic activity |
| preprobombyxin F1 | BAA19223.1 | 8.05E-45 | 1.285635 | receptor binding | chitin synthase A | NP_001245291.1 | 6.86E-05 | -1.125 | acetylglucosaminyltransferase activity |
| hypothetical protein KGM_15386 | EHJ78996.1 | 2.35E-07 | 1.254289 | one-carbon metabolic process | hypothetical protein KGM_14548 | EHJ75060.1 | 9.61E-11 | -1.14539 | ligase activity |
| ornithine decarboxylase | NP_001040457.1 | 2.08E-10 | 1.246224 | carboxy-lyase activity | angiotensin-converting enzyme | EHJ64693.1 | 5.66E-07 | -1.17124 | catalytic activity |
| cationic peptide CP8 precursor | ABL76064.1 | 1.69E-26 | 1.240764 | peptidase inhibitor activity | UDP-glycosyltransferase UGT41A3 precursor | NP_001243967.1 | 3.08E-09 | -1.28441 | catalytic activity |
| lysosomal thiol reductase IP30 isoform 2 precursor | NP_001103767.1 | 3.30E-05 | 1.212502 | catalytic activity | imaginal disk growth factor | BAF73623.1 | 8.77E-28 | -1.41966 | hydrolase activity, ion binding |
| PREDICTED: cullin-1 isoform 1 | XP_001606829.2 | 4.02E-25 | 1.195219 | enzyme binding | hypothetical protein KGM_21307 | EHJ68981.1 | 1.37E-10 | -1.61708 | lysine N-acetyltransferase activity |
| hypothetical protein KGM_20194 | EHJ77543.1 | 1.05E-09 | 1.165642 | nucleoside-triphosphatase activity | ATP-binding cassette sub-family B member 1 | ADV76536.1 | 1.19E-07 | -1.65133 | ATPase activity |
| GK14239 | XP_002073687.1 | 5.79E-79 | 1.146011 | NADH dehydrogenase activity | WW domain binding protein 4 | NP_001040408.1 | 1.75E-11 | -1.67674 | binding |
| s-adenosylmethionine decarboxylase | EHJ69734.1 | 5.26E-09 | 1.142828 | lyase activity | prophenoloxidase activating enzyme precursor | NP_001036832.1 | 1.77E-10 | -1.87357 | endopeptidase activity |
| hydroxypyruvate isomerase | ABY57912.1 | 7.26E-05 | 1.126609 | catalytic activity | antennal esterase CXE11 | AEJ38206.1 | 1.15E-07 | -1.9801 | catalytic activity |
| bombyxin B-7 precursor | NP_001121789.1 | 1.78E-125 | 1.115565 | receptor binding | hypothetical protein KGM_10315 | EHJ78878.1 | 6.30E-30 | -2.39866 | catalytic activity,binding |
| prepro-bombyxin D1'(K) | BAA20145.1 | 1.36E-124 | 1.066625 | receptor binding | integument esterase 2 precursor | NP_001121191.1 | 1.48E-05 | -3.04241 | catalytic activity |
| bombyxin B-1 precursor | NP_001121791.1 | 1.30E-05 | 1.027065 | receptor binding | hypothetical protein | BAC65324.1 | 4.19E-41 | -4.13436 | iron-sulfur cluster binding, hydro-lyase activity |
| **Cell component** | | | | | | | | | |
| **Name** | **Sequence ID** | **P-Value** | **log2** | **Ontology** | **Name** | **Sequence ID** | **P-Value** | **log2** | **Ontology** |
| muscle LIM protein isoform 1 | NP_001103762.1 | 9.52E-127 | 1.808547 | transition metal ion binding | putative BTB/POZ domain-containing protein KCTD9 | EHJ73113.1 | 4.54E-06 | -1.40541 | integral to plasma membrane |
| transmembrane protein precursor | NP_001040219.1 | 6.69E-08 | 1.200173 | intrinsic to membrane | Nesprin-1 | EFN84144.1 | 1.66E-14 | -1.55803 | intracellular organelle |
| vacuolar protein sorting 26 | NP_001040225.1 | 1.75E-10 | 1.08139 | endomembrane system | muscle-specific protein 300 | EHJ73088.1 | 1.43E-05 | -1.66118 | integral to membrane, cytoplasm |
| ARP1 actin-related protein 1-like protein A | NP_001040336.1 | 1.83E-34 | 1.044463 | actin-related protein | putative formin 1,2/cappuccino | EHJ71733.1 | 7.60E-05 | -1.85866 | cytoplasmic part |
| **Transport** | | | | | | | | | |
| **Name** | **Sequence ID** | **P-Value** | **log2** | **Ontology** | **Name** | **Sequence ID** | **P-Value** | **log2** | **Ontology** |
| trafficking protein particle complex subunit 4 | ADO95152.1 | 2.35E-09 | 1.684421 | Golgi vesicle transport | coatomer protein complex subunit alpha | NP_001166192.1 | 1.43E-08 | -1.40824 | binding,transferase activity |
| transmembrane emp24 protein transport domain containing 9 precursor | NP_001040538.1 | 2.68E-29 | 1.010151 | regionalization | PREDICTED: similar to mannosyl-oligosaccharide 1,2-alpha-mannosidase IB | XP_973062.1 | 7.21E-08 | -2.00329 | metal ion binding |
| coatomer protein complex subunit beta | NP_001166193.1 | 1.59E-13 | -1.07794 | protein transport |  |  |  |  |  |
| **Transcription** | | | | | | | | | |
| **Name** | **Sequence ID** | **P-Value** | **log2** | **Ontology** | **Name** | **Sequence ID** | **P-Value** | **log2** | **Ontology** |
| troponin I transcript variant C | ACN86370.1 | 1.22E-17 | 1.92714 | transcription | putative ebna2 binding protein P100 | EHJ72857.1 | 1.65E-14 | -1.48023 | nucleic acid binding |
| putative RNA polymerase III polypeptide H | EHJ76803.1 | 5.70E-12 | 1.1574 | RNA polymerase activity | mediator of RNA polymerase II transcription subunit 29 | NP_001037105.1 | 8.85E-10 | -1.61326 | transcription |
| hypothetical protein KGM_13528 | EHJ73992.1 | 4.31E-06 | 1.019388 | RNA binding | transcription initiation factor IIE subunit beta | EHJ64456.1 | 8.20E-19 | -1.71932 | transcription |
| ap endonuclease | EHJ69244.1 | 2.86E-12 | -1.12018 | nuclease activity | AGAP001234-PA | XP_321922.5 | 4.43E-59 | -1.99505 | nucleoside-triphosphatase activity, nucleotide binding |
| transcription initiation factor TFIID subunit 10 | EHJ74593.1 | 3.82E-06 | -1.28812 | translation factor activity, nucleic acid binding | hypothetical protein SINV_04027 | EFZ12014.1 | 8.19E-07 | -2.48618 | alternative nuclear mRNA splicing, via spliceosome |
| putative UPF2 regulator of nonsense transcripts-like protein | EHJ76147.1 | 8.77E-11 | -1.38203 | transcription | putative ribosomal RNA methyltransferase | EHJ67114.1 | 2.25E-11 | -1.50709 | transcription |
| **Translation** | | | | | | | | | |
| **Name** | **Sequence ID** | **P-Value** | **log2** | **Ontology** | **Name** | **Sequence ID** | **P-Value** | **log2** | **Ontology** |
| PREDICTED: u6 snRNA-associated Sm-like protein LSm5-like | XP_001951255.1 | 1.11E-112 | 1.581535 | Gbinding | ribosomal protein P1 | NP_001091750.1 | 3.06E-09 | 1.594065 | translation |
| eukaryotic translation initiation factor 3 subunit I | NP_001040433.1 | 8.87E-70 | 1.478237 | translation factor activity, nucleic acid binding | ribosomal protein S12 | NP_001037568.1 | 7.08E-85 | 1.166792 | structural molecule activity |
| eukaryotic translation initiation factor 3 subunit H | NP_001036848.1 | 8.52E-98 | 1.476724 | translation factor activity, nucleic acid binding | ribosomal protein S6 | NP_001037566.1 | 3.66E-138 | 1.105528 | structural molecule activity |
| eukaryotic translation initiation factor 3 subunit F | NP_001040528.1 | 3.70E-65 | 1.406752 | translation factor activity, nucleic acid binding | 28S ribosomal protein S6 precursor | EHJ72084.1 | 1.33E-07 | 1.067408 | structural molecule activity |
| elongation factor 1 alpha | AFJ44727.1 | 0 | 1.367387 | nucleoside-triphosphatase activity | 60S ribosomal protein L18 | NP_001037217.1 | 2.50E-277 | 1.000449 | structural molecule activity |
| eukaryotic translation initiation factor 4A | ABF51379.1 | 5.73E-190 | 1.306995 | translation factor activity, nucleic acid binding | ribo nucleo protein | ADO33044.1 | 1.60E-16 | -1.30003 | structural molecule activity |
| hypothetical protein KGM_20795 | EHJ75707.1 | 2.81E-05 | 1.237333 | RNA binding, transition metal ion binding | putative aminoacyl-tRNA synthetase | EHJ77748.1 | 1.01E-51 | -1.64985 | aminoacyl-tRNA ligase activity |
| eukaryotic translation termination factor 1 | NP_001040243.1 | 1.94E-08 | 1.076878 | translation release factor activity | sensitized chromosome inheritance modifier 19 | EHJ72595.1 | 2.27E-07 | -2.34694 | translation |
| ribosomal protein S7 | NP_001037261.1 | 9.41E-221 | 1.653154 | structural molecule activity |  |  |  |  |  |
| **Growth and development** | | | | | | | | | |
| **Name** | **Sequence ID** | **P-Value** | **log2** | **Ontology** | **Name** | **Sequence ID** | **P-Value** | **log2** | **Ontology** |
| hypothetical protein KGM_05708 | EHJ68237.1 | 1.26E-14 | 2.093543 | cytoskeletal protein binding | hypothetical protein KGM_14760 | EHJ77437.1 | 4.68E-09 | -1.25428 | protein binding, respiratory system development |
| chemosensory protein 7 precursor | NP_001037068.1 | 3.61E-32 | 1.487136 | organ development | wing disc-specific protein | NP_001036961.1 | 4.74E-14 | -1.31716 | organ development |
| hemolymph juvenile hormone binding protein precursor | AAF19267.1/AF098304_1 | 5.20E-05 | 1.430674 | juvenile hormone associated | sex-specific storage-protein 2 precursor | NP_001037590.1 | 2.88E-176 | -1.50771 | sex-specific protein |
| hypothetical protein KGM_08051 | EHJ72760.1 | 1.95E-09 | 1.138445 | respiratory system development | odorant binding protein LOC100307012 precursor | NP_001159621.1 | 7.83E-05 | -1.93119 | odorant binding |
| presenilin-like signal peptide peptidase | NP_001040306.1 | 4.05E-09 | 1.130913 | aspartic-type endopeptidase activity | silkworm storage protein | AFD02109.1 | 6.44E-06 | -2.3374 | tissue development |
| selenophosphate synthetase 1 | NP_001037388.1 | 2.81E-10 | 1 | kinase activity,organ development | muscle myosin heavy chain | BAG30740.1 | 8.50E-25 | -3.65631 | identical protein binding, motor activity |
| receptor guanylyl cyclase GC-II | AAN16469.1 | 1.93E-11 | -1.25407 | cyclase activity, lyase activity | odorant binding protein | BAH79159.1 | 1.66E-12 | -3.73789 | odorant binding |
| **Signal transduction** | | | | | | | | | |
| **Name** | **Sequence ID** | **P-Value** | **log2** | **Ontology** | **Name** | **Sequence ID** | **P-Value** | **log2** | **Ontology** |
| putative tubulin folding cofactor E | EHJ77807.1 | 1.70E-19 | 1.498191 | synaptic transmission | vacuolar ATP synthase subunit E | NP_001040451.1 | 9.24E-05 | 0.547473 | hydrogen ion transmembrane transporter activity |
| H+ transporting ATP synthase gamma subunit | NP_001040428.1 | 4.39E-39 | 1.324139 | hydrogen ion transmembrane transporter activity | acetylcholinesterase type 1 | ABY50088.1 | 0.694122 | 0.067534 | acetylcholine catabolic process |
| PREDICTED: similar to sprouty | XP_973145.2 | 6.11E-06 | 1.255704 | transmembrane receptor protein tyrosine kinase signaling pathway | putative Insulin receptor precursor | EHJ69301.1 | 2.51E-07 | -1.00616 | signal transducer activity, protein kinase activity |
| allatostatin C precursor | NP_001124356.1 | 1.38E-32 | 1.127202 | G-protein coupled receptor protein signaling pathway | putative BTB/POZ domain-containing protein KCTD9 | EHJ73113.1 | 4.54E-06 | -1.40541 | potassium channel activity |
| CHH-like protein precursor | NP_001106139.1 | 2.51E-07 | 1.047027 | G-protein coupled receptor protein signaling pathway | neuropeptide receptor B3 precursor | NP_001127734.1 | 1.88E-05 | -1.89998 | G-protein coupled receptor activity |
| Ssu72 domain protein | ACM24350.1 | 2.17E-09 | 1.024404 | signal transducer activity, phosphatase activity | putative Spectrin alpha chain | EHJ74635.1 | 2.56E-22 | -2.32409 | tubulin binding, regulation of synaptic growth at neuromuscular junction |
| **Immune response** | | | | | | | | | |
| **Name** | **Sequence ID** | **P-Value** | **log2** | **Ontology** | **Name** | **Sequence ID** | **P-Value** | **log2** | **Ontology** |
| apolipophorin III precursor | NP_001037078.1 | 3.32E-87 | 1.645729 | binding, defense response to bacterium | cuticular protein RR-2 motif 65 precursor | NP_001166692.1 | 7.77E-08 | -3.46983 | cuticular protein RR-2 motif 65 precursor |
| 30K protein 7 | AFC87805.1 | 1.18E-16 | 2.681908 | immune response | 30kDa protein | ADQ89805.1 | 2.50E-42 | -3.2114 | immune response |
| phosphatidylethanolamine binding protein isoform 1 | NP_001166821.1 | 3.77E-19 | 2.388807 | immune response |  |  |  |  |  |
| **Cell cycle** | | | | | | | | | |
| **Name** | **Sequence ID** | **P-Value** | **log2** | **Ontology** | **Name** | **Sequence ID** | **P-Value** | **log2** | **Ontology** |
| putative cyclin | EHJ63808.1 | 6.51E-12 | 1.055843 | cell cycle | rothmund-thomson syndrome DNA helicase recq4 | EHJ75407.1 | 1.90E-06 | -1.42864 | double-stranded DNA binding |
| meiotic recombination 11 | NP_001036845.1 | 1.61E-07 | -1.01436 | transition metal ion binding | DNA helicase recq5 | EHJ77194.1 | 0.00011916 | -1.82 | ATP-dependent DNA helicase activity |
| hypothetical protein KGM_15729 | EHJ71746.1 | 9.88E-11 | -1.13381 | nucleic acid binding, endonuclease activity | alpha-tubulin | ABU94676.1 | 0 | -2.3471 | structural molecule activity, nucleoside-triphosphatase activity |
| hypothetical protein KGM_14990 | EHJ66282.1 | 1.22E-11 | -1.31576 | regulation of cell cycle proce |  |  |  |  |  |
| **Apoptosis** |  |  |  |  |  |  |  |  |  |
| **Name** | **Sequence ID** | **P-Value** | **log2** | **Ontology** | **Name** | **Sequence ID** | **P-Value** | **log2** | **Ontology** |
| elongation factor 1 gamma | NP_001036852.1 | 9.15E-23 | 1.313124 | translation factor activity, | cytochrome c oxidase polypeptide IV | NP_001073120.1 | 4.38E-80 | 1.166319 | heme-copper terminal oxidase activity |
| cytochrome c oxidase polypeptide Vb | NP_001106742.1 | 1.81E-42 | 1.30167 | heme-copper terminal oxidase activity | mitochondrial cytochrome c | ACF41193.1 | 1.42E-24 | -0.08463 | electron carrier activity |
| hypothetical protein KGM_05439 | EHJ65404.1 | 8.34E-54 | 1.209147 | quinone binding, catalytic activity | putative Sentrin-specific protease | EHJ77080.1 | 1.06E-05 | -1.76802 | small conjugating protein-specific protease activity |
