# Supplementary material for: Molecular Mechanisms of Reduced Nerve Toxicity by Titanium Dioxide Nanoparticles in the Phoxim-Exposed Brain of Bombyx mori
Source: PLoS One. 2014 Jun 27;9(6):e101062. doi: 10.1371/journal.pone.0101062 (PMC4074129; doi:10.1371/journal.pone.0101062)
Supplement: Table S3 — Genes related to oxidative stress, stress response, metabolic process, cell component, transport, transcription, translation, growth and development, signal transduction, immune response, cell cycle and apoptosis altered significantly by TiO2 NPs + phoxim exposure. (DOC) [file pone.0101062.s006.doc]

**Table S3** Genes related to oxidative stress, stress response, metabolic process, cell component, transport, transcription, translation, growth and development, signal transduction, immune response, cell cycle and apoptosis altered significantly by TiO_2_ NPs + phoxim exposure.

| **Oxidative stress** |  |  |  |  |  |  |  |  |  |
| --- | --- | --- | --- | --- | --- | --- | --- | --- | --- |
| **Name** | **Sequence ID** | **P-Value** | **log2** | **Ontology** | **Name** | **Sequence ID** | **P-Value** | **log2** | **Ontology** |
| Hypothetical protein KGM_11849 | EHJ78758.1 | 2.10E-12 | 4.081388 | oxidoreductase activity | superoxide dismutase | NP_001037084.1 | 5.59E-06 | -0.471544 | catalytic activity |
| NADH dehydrogenase | NP_001040478.1 | 8.59E-25 | -1.1007 | oxidoreductase activity | hypothetical protein KGM_05873 | EHJ77149.1 | 3.76E-05 | -1.7339 | oxidoreductase activity |
| dihydropyrimidine dehydrogenase | EHJ74888.1 | 4.81E-32 | -1.10121 | oxidoreductase activityr | hydroxysteroid(17-beta)dehydrogenase4 | EFA07495.1 | 4.52E-25 | -1.78706 | catalytic activity |
| ferritin precursor | NP_001037580.1 | 5.87E-56 | -1.10956 | oxidoreductase activity | chymotrypsin inhibitor CI-8A | AAK52495.1/AF361483_1 | 9.27E-09 | -1.88753 | endopeptidase inhibitor activity |
| dihydrolipoamide dehydrogenase | NP_001037054.1 | 9.41E-06 | -1.39022 | oxidoreductase activity | pyridoxine 5'-phosphate oxidase | NP_001037442.1 | 1.49E-10 | -1.97265 | oxidoreductase activity |
| cytochrome P450 9a20 | NP_001077079.1 | 2.24E-15 | -1.42949 | oxidoreductase activity | putative alcohol dehydrogenase | EHJ73729.1 | 5.92E-10 | -2.03428 | catalytic activity |
| TATA-box-binding protein | NP_001037059.1 | 0.0001173 | -1.44478 | oxidoreductase activity | short-chain dehydrogenease/reductase | EHJ73302.1 | 1.39E-18 | -2.09712 | catalytic activity |
| glutamate dehydrogenase | NP_001040245.1 | 6.30E-06 | -1.4828 | oxidoreductase activity | lactate dehydrogenase | NP_001095933.1 | 5.20E-06 | -2.48631 | lactate dehydrogenase activity |
| desaturase | AAQ74260.1 | 2.99E-14 | -1.48781 | oxidoreductase activity | hypothetical protein | BAC65324.1 | 7.30E-32 | -3.00451 | iron-sulfur cluster binding,hydro-lyase activity |
| short-chain dehydrogenase | EHJ65140.1 | 9.41E-07 | -1.61033 | catalytic activity | vesicle amine transport protein | NP_001093281.1 | 7.66E-22 | -4.17544 | oxidoreductase activity |
| putative mitochondrial NADH-ubiquinone oxidoreductase AGGG subunit | EHJ75647.1 | 2.14E-41 | -1.70888 | catalytic activity | cytosolic malate dehydrogenase | NP_001040257.1 | 1.10E-05 | -8.88874 | Malate dehydrogenase activity |
| dopa-decarboxylase | AAR23825.1 | 3.28E-06 | -1.71277 | carboxy-lyase activity | triosephosphate isomerase | NP_001119730.1 | 5.59E-06 | -8.97154 | intramolecular oxidoreductase activity |
| thiol peroxiredoxin | NP_001037083.1 | 4.50E-38 | -0.40615 | oxidoreductase activity |  |  |  |  |  |
| **Stress response** | | | | | | | | | |
| **Name** | **Sequence ID** | **P-Value** | **log2** | **Ontology** | **Name** | **Sequence ID** | **P-Value** | **log2** | **Ontology** |
| heat shock cognate protein | NP_001036892.1 | 8.57E-29 | 0.220783 | response to stress | DnaJ (Hsp40) homolog 3 | NP_001157381.1 | 9.80E-19 | -1.96708 | protein binding |
| heat shock cognate 70 protein | EHJ73638.1 | 3.62E-13 | -1.28028 | lyase activity | heat shock protein 25.4 precursor | NP_001112375.1 | 6.28E-07 | -3.03076 | response to stimulus |
| 19.8 kDa small heat shock protein | EHJ68318.1 | 4.29E-103 | -1.49372 | response to stimulus | Peptidoglycan recognition protein precursor | NP_001036836.1 | 1.69E-09 | -3.17544 | hydrolase activity |
| DnaJ-19 | AFC01233.1 | 3.38E-08 | -1.79582 | protein binding |  |  |  |  |  |
| **Metabolic process** | | | | | | | | | |
| **Name** | **Sequence ID** | **P-Value** | **log2** | **Ontology** | **Name** | **Sequence ID** | **P-Value** | **log2** | **Ontology** |
| putative dual specificity phosphatase 23-like protein | EHJ77432.1 | 1.22E-07 | 3.467217 | phosphatase activity | adenylate kinase 2 | NP_001165387.1 | 7.04E-07 | -1.544661 | nucleobase, nucleoside, nucleotide kinase activity |
| hypothetical protein KGM_19988 | EHJ76797.1 | 2.32E-31 | 1.708091 | phosphotransferase activity | uridine phosphorylase | EHJ70429.1 | 3.52E-06 | -1.569905 | catalytic activity |
| prepro-bombyxin D1'(K) | BAA20145.1 | 1.54E-145 | 1.138202 | receptor binding | peptidylprolyl isomerase B precursor | NP_001040479.1 | 1.16E-08 | -1.604158 | cis-trans isomerase activity |
| hypothetical protein KGM_01171 | EHJ77400.1 | 4.90E-06 | -1.01342 | ATPase activity | PREDICTED: serine/threonine-protein kinase tousled-like 2-like | XP_003690963.1 | 1.00E-07 | -1.659584 | protein kinase activity |
| Bmsqd-2 | BAA07211.1 | 5.07E-05 | -1.01772 | binding | hypothetical protein KGM_01156 | EHJ66405.1 | 1.65E-07 | -1.664783 | protein kinase activity |
| hypothetical protein KGM_04824 | EHJ68794.1 | 3.57E-26 | -1.02353 | hydrolase activity, | insulin-related peptide binding protein precursor | NP_001239349.1 | 1.08E-07 | -1.68373 | catalytic activity |
| hypothetical protein KGM_01435 | EHJ78139.1 | 2.32E-06 | -1.0348 | endopeptidase activity | proteasome subunit beta 5 | EHJ72628.1 | 5.92E-21 | -1.686317 | endopeptidase activity |
| putative high mobility group 20A isoform 1 | EHJ71875.1 | 6.90E-16 | -1.04324 | binding | putative DNA replication complex GINS protein pSF2 | EHJ68662.1 | 3.49E-05 | -1.690015 | DNA metabolic process |
| hypothetical protein KGM_15891 | EHJ66129.1 | 8.11E-05 | -1.14461 | endonuclease activity | Cullin-3 | EFN80806.1 | 2.85E-19 | -1.705225 | enzyme binding, small conjugating protein ligase activity |
| putative DNA-directed RNA polymerase II 13.3 kDa polypeptide | EHJ65777.1 | 6.48E-07 | -1.24305 | RNA polymerase activity, | LSM Sm-like protein family member | NP_001040418.1 | 1.82E-12 | -1.777844 | RNA binding |
| hypothetical protein KGM_15103 | EHJ75587.1 | 9.27E-06 | -1.246 | iron-sulfur cluster binding | asparagine synthetase | NP_001037414.1 | 1.85E-26 | -1.790323 | carbon-nitrogen ligase activit |
| imaginal disk growth factor | BAF73623.1 | 2.45E-23 | -1.2546 | hydrolase activity, | hypothetical protein KGM_03040 | EHJ67089.1 | 8.16E-18 | -1.832436 | cellular protein metabolic process |
| hypothetical protein KGM_15589 | EHJ79128.1 | 7.73E-32 | -1.26197 | ATPase activity, coupled | putative chromatin regulatory protein sir2 | EHJ73249.1 | 5.29E-11 | -1.83373 | binding, primary metabolic process |
| glyceraldehyde-3-phosphate dehydrogenase | BAE96011.1 | 4.39E-05 | -1.26289 | nucleotide binding | proteasome subunit alpha type 6-A | NP_001040459.1 | 2.23E-15 | -1.883241 | endopeptidase activity |
| ubiquitin-conjugating enzyme E2M | NP_001040241.1 | 1.52E-21 | -1.28699 | small conjugating protein ligase activity | putative protein arginine N-methyltransferase | EHJ76958.1 | 2.22E-34 | -1.986877 | protein-arginine N-methyltransferase activity |
| receptor guanylyl cyclase GC-II | AAN16469.1 | 4.50E-12 | -1.30164 | cyclase activity, | hypothetical protein DAPPUDRAFT_41314 | EFX88542.1 | 6.76E-06 | -2.046503 | transaminase activity, cofactor binding |
| angiotensin-converting enzyme | EHJ64693.1 | 1.64E-08 | -1.36445 | metabolic process | hypothetical protein KGM_02567 | EHJ67837.1 | 2.51E-07 | -2.049834 | ligase activity, nucleotide binding |
| proteasome subunit beta 7 | NP_001040536.1 | 2.39E-36 | -1.37835 | endopeptidase activity | dolichyl-phosphate mannosyltransferase | NP_001040468.1 | 1.27E-34 | -2.05203 | transferase activity |
| hypothetical protein TcasGA2_TC007844 | EFA02188.1 | 4.20E-31 | -1.39412 | hydrolase activity | AGAP006670-PA | XP_316706.4 | 5.68E-15 | -2.05999 | hydrolase activity |
| hypothetical protein KGM_05863 | EHJ77133.1 | 2.92E-05 | -1.39437 | ATPase activity, coupled | prp4 | EHJ66330.1 | 3.97E-07 | -2.085511 | kinase activity |
| hypothetical protein KGM_20645 | EHJ71883.1 | 2.92E-05 | -1.39437 | cellular metabolic process | hypothetical protein KGM_11399 | EHJ65339.1 | 1.21E-49 | -2.143855 | endopeptidase activity |
| hypothetical protein KGM_20562 | EHJ76460.1 | 1.25E-39 | -1.43219 | transition metal ion binding | calreticulin | BAC57964.1 | 1.94E-15 | -2.170986 | metal ion binding, carbohydrate binding |
| S-adenosyl-L-homocysteine hydrolase | NP_001093271.1 | 2.26E-05 | -1.43522 | cellular metabolic process | PREDICTED: similar to widerborst CG5643-PC | XP_971164.2 | 1.82E-16 | -2.197066 | phosphoprotein phosphatase activity |
| replication factor C4 | NP_001040483.1 | 7.29E-16 | -1.44924 | DNA binding | DNA-damage inducible protein | NP_001040314.1 | 3.63E-07 | -2.235833 | endopeptidase activity |
| fructose-1,6-bisphosphatase | NP_001040381.1 | 1.28E-19 | -1.45022 | carbohydrate phosphatase activity | hypothetical protein KGM_10825 | EHJ65868.1 | 6.21E-12 | -2.334928 | transaminase activity |
| male reproductive organ angiotensin-converting enzyme-related protein 2 | NP_001153676.1 | 1.16E-09 | -1.49061 | exopeptidase activity | beta-hexosaminidase | ABO65045.1 | 2.09E-06 | -2.4208 | hydrolase activity |
| proteasome 25 kDa subunit | NP_001040344.1 | 1.43E-37 | -1.49915 | endopeptidase activity | phosphotriesterase-like protein | NP_001185957.1 | 3.72E-10 | -2.423563 | hydrolase activity |

| C-type lectin 11 precursor | NP_001037076.1 | 1.94E-05 | -1.50309 | carbohydrate binding | AGAP002409-PA | XP_312543.3 | 9.30E-05 | -2.587034 | RNA metabolic process |
| --- | --- | --- | --- | --- | --- | --- | --- | --- | --- |
| ribosomal protein S27a | NP_001091826.1 | 6.64E-301 | -1.50688 | transaminase activity | isocitrate dehydrogenase | NP_001040134.1 | 5.42E-05 | -2.643856 | metal ion binding |
| annexin isoform 2 | NP_001091759.1 | 7.86E-06 | -1.52541 | phospholipid binding | 26S protease regulatory subunit 6A | EHJ68433.1 | 1.21E-25 | -2.878083 | nucleoside-triphosphatase activity |
| hypothetical protein KGM_08223 | EHJ66459.1 | 1.05E-06 | -1.52546 | cis-trans isomerase activity | hypothetical protein KGM_22527 | EHJ67117.1 | 5.38E-05 | -3.334312 | metabolic process |
| hypothetical protein KGM_21462 | EHJ65222.1 | 3.16E-08 | -1.52579 | intramolecular oxidoreductase activity | short-chain dehydrogenease/reductase-like | NP_001040155.1 | 3.17E-10 | -3.49737 | catalytic activity |
| phosphatidylinositol glycan precursor | NP_001040502.1 | 5.99E-05 | -1.52725 | endopeptidase activity | cationic peptide CP8 precursor | ABL76064.1 | 1.09E-53 | -5.614964 | peptidase inhibitor activity |
| **Cell component** |  |  |  |  |  |  |  |  |  |
| **Name** | **Sequence ID** | **P-Value** | **log2** | **Ontology** | **Name** | **Sequence ID** | **P-Value** | **log2** | **Ontology** |
| hypothetical protein KGM_05708 | EHJ68237.1 | 3.38E-05 | 1.296552 | cytoskeletal protein binding | hypothetical protein KGM_22198 | EHJ65119.1 | 3.01E-14 | -1.308535 | intrinsic to membrane |
| muscle myosin heavy chain | BAG30740.1 | 2.23E-15 | 1.213915 | identical protein binding | PIN2/TRF1-interacting protein | EHJ63380.1 | 1.05E-10 | -1.359996 | binding |
| ARP1 actin-related protein 1-like protein A | NP_001040336.1 | 7.95E-17 | -1.01766 | actin-related protein | coatomer protein complex subunit zeta | ADB66739.1 | 8.73E-10 | -1.847307 | establishment of localization |
| hypothetical protein KGM_08440 | EHJ67977.1 | 2.49E-19 | -1.05152 | cytoplasmic part | hypothetical protein KGM_04332 | EHJ69967.1 | 1.74E-05 | -2.05823 | cell part |
| mRNA transport regulator 3 | NP_001093283.1 | 5.61E-06 | -1.16373 | nucleic acid binding | hypothetical protein KGM_06005 | EHJ66248.1 | 5.55E-07 | -2.296275 | cell part |
| hypothetical protein KGM_11118 | EHJ69096.1 | 1.86E-11 | -1.18255 | microtubule cytoskeleton | transmembrane trafficking protein precursor | NP_001040510.1 | 8.70E-27 | -2.674784 | intrinsic to membrane |
| Mps one binder kinase activator-like 4 | EFN65561.1 | 1.10E-12 | -1.20705 | microtubule organizing center |  |  |  |  |  |
| **Transport** |  |  |  |  |  |  |  |  |  |
| **Name** | **Sequence ID** | **P-Value** | **log2** | **Ontology** | **Name** | **Sequence ID** | **P-Value** | **log2** | **Ontology** |
| putative Aktip protein | EHJ76935.1 | 1.85E-10 | -1.04614 | lysosomal transport, endosome transport | vesicle protein sorting-associated | XP_001654152.1 | 3.69E-17 | -1.477282 | protein binding,  protein transpor |
| globin 1 | NP_001136083.1 | 6.71E-13 | -1.17549 | iron ion binding,  gas transport | nuclear migration protein nudC | NP_001040262.1 | 8.36E-48 | -1.490605 | transport |
| hypothetical protein KGM_14685 | EHJ77957.1 | 7.89E-05 | -1.17632 | amine transmembrane transporter activity | sugar transporter | EHJ73890.1 | 3.02E-08 | -1.738477 | transport |
| hypothetical protein KGM_15746 | EHJ71712.1 | 1.88E-05 | -1.29215 | phosphotransferase activity | vacuolar ATP synthase subunit G | NP_001040287.1 | 1.73E-22 | -2.19752 | transport |
| hypothetical protein KGM_04765 | EHJ73963.1 | 2.48E-05 | -1.31529 | transferase activity | putative sugar transporter | EHJ70460.1 | 2.48E-05 | -2.357956 | transport |
| hypothetical protein KGM_04631 | EHJ77618.1 | 2.84E-07 | -1.37106 | metal ion binding | vesicular inhibitory amino acid transporter | EHJ68677.1 | 6.23E-09 | -2.453994 | transport |
| membrane protein TMS1 precursor | NP_001037624.1 | 1.13E-12 | -1.42969 | transport |  |  |  |  |  |
| **Transcription** |  |  |  |  |  |  |  |  |  |
| **Name** | **Sequence ID** | **P-Value** | **log2** | **Ontology** | **Name** | **Sequence ID** | **P-Value** | **log2** | **Ontology** |
| PREDICTED: tRNA pseudouridine synthase 3-like | XP_001602396.1 | 2.46E-13 | 1.522941 | RNA modification | hypothetical protein KGM_02279 | EHJ74878.1 | 1.80E-15 | -1.687182 | RNA helicase activity, ATPase activity, coupled |
| putative nuclear transcription factor, X-box binding protein | EHJ72907.1 | 1.69E-07 | -1.02529 | transcription | putative pom1 | EHJ66101.1 | 2.75E-15 | -1.776524 | DNA helicase activity, structure-specific DNA binding |
| elongation protein 4-like protein | NP_001040120.1 | 5.19E-05 | -1.05686 | nuclear mRNA splicing | splicing factor 45 | ABG42998.1 | 3.73E-06 | -1.790676 | binding |
| hypothetical protein KGM_10301 | EHJ78848.1 | 1.10E-05 | -1.09057 | binding | putative RNA polymerase II associated protein 2 | EHJ73636.1 | 0.000117 | -2.089552 | transcription |
| hypothetical protein KGM_14516 | EHJ76983.1 | 7.29E-06 | -1.20112 | nucleic acid binding | oligoribonuclease, mitochondrial | EHJ78664.1 | 4.39E-05 | -2.180154 | exonuclease activity |
| AGAP001234-PA | XP_321922.5 | 2.61E-29 | -1.20436 | nucleoside-triphosphatase activity | Heterogeneous nuclear ribonucleoprotein K | EHJ63796.1 | 6.73E-37 | -2.288753 | nucleic acid binding |
| transcription initiation factor IIE subunit beta | EHJ64456.1 | 3.22E-12 | -1.24262 | transcription | exosc7 protein | NP_001040497.1 | 5.86E-06 | -2.344493 | nucleic acid binding |
| hypothetical protein KGM_04294 | EHJ72693.1 | 4.10E-07 | -1.35604 | nucleoside-triphosphatase activity | transcription initiation factor TFIID subunit 12 | NP_001040422.1 | 2.01E-07 | -2.357956 | translation factor activity, nucleic acid binding |
| hypothetical protein KGM_02320 | EHJ74315.1 | 9.41E-07 | -1.5071 | RNA polymerase activity, nucleic acid binding, nucleoside binding | mediator of RNA polymerase II transcription subunit 29 | NP_001037105.1 | 7.80E-21 | -3.251494 | transcription |
| putative Forkhead box protein E1 | EHJ71148.1 | 7.84E-49 | -1.5115 | DNA binding | splicing factor proline- and glutamine-rich | ABG43001.1 | 1.07E-08 | -4.071277 | nucleic acid binding |
| endothelial-monocyte activating polypeptide II | NP_001040121.1 | 5.53E-14 | -1.57912 | RNA binding | transcription initiation factor TFIID subunit 10 | EHJ74593.1 | 3.73E-23 | -5.385788 | translation factor activity, nucleic acid binding |
| cleavage and polyadenylation specific factor 4 | NP_001040511.1 | 6.78E-07 | -1.65648 | transition metal ion binding |  |  |  |  |  |
| **Translation** |  |  |  |  |  |  |  |  |  |
| **Name** | **Sequence ID** | **P-Value** | **log2** | **Ontology** | **Name** | **Sequence ID** | **P-Value** | **log2** | **Ontology** |
| putative tRNA splicing endonuclease 54-like protein | EHJ68012.1 | 3.64E-10 | 1.336249 | translation | ribosomal protein L7A | NP_001037138.1 | 5.06E-159 | -1.383088 | ribonucleoprotein complex biogenesis |
| ribosomal protein L20 | NP_001040151.1 | 9.33E-22 | 1.333733 | RNA binding | hypothetical protein KGM_08001 | EHJ69245.1 | 2.40E-08 | -1.391047 | aminoacyl-tRNA ligase activity |
| 28S ribosomal protein S6 precursor | EHJ72084.1 | 7.47E-12 | 1.322934 | structural molecule activity | ribosomal protein S20 | NP_001091809.1 | 3.71E-288 | -1.459461 | structural molecule activity |
| putative adenosine deaminase acting on tRNA | EHJ76545.1 | 2.38E-06 | 1.013874 | adenosine deaminase activity | elongation factor Tu | NP_001040119.1 | 3.64E-28 | -1.534103 | nucleoside-triphosphatase activity |
| putative aminoacyl-tRNA synthetase | EHJ77748.1 | 3.14E-25 | -1.01075 | aminoacyl-tRNA ligase activity | uncharacterized protein LOC767623 | NP_001091746.1 | 5.27E-06 | -1.548234 | binding |
| putative 39S ribosomal protein L15, mitochondrial | EHJ64516.1 | 1.25E-06 | -1.02845 | structural molecule activity | mitochondrial ribosomal protein S10 | NP_001040362.1 | 1.17E-05 | -1.636698 | structural molecule activity |
| lysyl-tRNA synthetase | AEB77710.1 | 2.61E-11 | -1.05777 | aminoacyl-tRNA ligase activity | translin | NP_001040282.1 | 8.68E-12 | -1.864466 | endonuclease activity, DNA binding |
| ribosomal protein L7 | NP_001037135.1 | 9.48E-16 | -1.07136 | structural molecule activity | mitochondrial ribosomal protein, L55, putative | XP_001651805.1 | 2.27E-12 | -1.903002 | nucleic acid binding, structural molecule activity |
| mitochondrial ribosomal protein L54 | NP_001098690.1 | 1.48E-07 | -1.10083 | structural molecule activity | mitochondrial ribosomal protein S5 | NP_001040341.1 | 5.88E-15 | -1.941076 | nucleic acid binding |
| ribosomal protein S24 | ABS57445.1 | 5.89E-254 | -1.10186 | structural molecule activity, binding | hypothetical protein KGM_15844 | EHJ72864.1 | 5.54E-34 | -1.950212 | protein methyltransferase activity |
| eukaryotic initiation factor 4A-III | NP_001106217.1 | 1.01E-18 | -1.1282 | ATPase activity, RNA helicase activity | putative tyrosyl-tRNA synthetase | EHJ76165.1 | 6.04E-09 | -2.025381 | aminoacyl-tRNA ligase activity |
| elongation factor Ts | NP_001040359.1 | 9.54E-13 | -1.20457 | translation factor activity | ribosomal protein L26 | NP_001037233.1 | 5.54E-21 | -2.272001 | structural molecule activity |
| elongation factor 1 delta | NP_001036853.1 | 4.28E-56 | -1.25368 | translation factor activity | small nuclear ribonucleoprotein protein F | NP_001093276.1 | 2.09E-06 | -2.4208 | binding |
| ribosomal protein L27A | NP_001037522.1 | 8.07E-10 | -1.3084 | structural molecule activity | putative ribosome biogenesis protein bop1 | EHJ71369.1 | 8.18E-21 | -2.624619 | ribonucleoprotein complex biogenesis |
| phenylalanyl-tRNA synthetase beta subunit | NP_001182135.1 | 4.10E-07 | -1.35604 | aminoacyl-tRNA ligase activity | hypothetical protein AND_17160 | EFR21355.1 | 4.76E-28 | -3.190506 | aminoacyl-tRNA ligase activity |
| ribosomal protein L37 | NP_001037247.1 | 1.95E-10 | -1.36306 | RNA binding | GF12641 | XP_001958961.1 | 3.06E-10 | -3.8168 | translation |
| eukaryotic translation initiation factor 3 subunit E | NP_001037404.1 | 4.09E-41 | -1.38093 | translation factor activity |  |  |  |  |  |
| **Growth and development** | | | | | | | | | |
| **Name** | **Sequence ID** | **P-Value** | **log2** | **Ontology** | **Name** | **Sequence ID** | **P-Value** | **log2** | **Ontology** |
| miniparamyosin | ACM17460.1 | 1.13E-16 | 1.116454 | tissue development | structural maintenance of chromosomes 1A | EHJ76633.1 | 6.75E-06 | -1.974909 | organelle organization, brain development |
| muscle LIM protein isoform 1 | NP_001103762.1 | 9.70E-36 | 1.071296 | anatomical structure development | odorant binding protein | BAH36759.1 | 1.11E-66 | -2.56366 | odorant binding |
| hypothetical protein KGM_14747 | EHJ72562.1 | 3.26E-05 | -1.06995 | growth factor receptor binding | GE11757 | XP_002092274.1 | 5.42E-05 | -2.643856 | regulation of developmental growth |
| hypothetical protein KGM_05415 | EHJ68423.1 | 6.15E-05 | -1.34761 | embryonic development | silkworm storage protein | AFD02109.1 | 2.12E-08 | -3.566885 | tissue development |
| putative ecdysone oxidase | EHJ63852.1 | 8.17E-11 | -1.34772 | ecdysone associated | hypothetical protein KGM_00312 | EHJ76256.1 | 2.90E-11 | -3.61471 | skeletal muscle fiber development |
| myocyte enhancing factor 2 isoform A | NP_001036905.1 | 1.14E-07 | -1.49456 | midgut development | mobility group protein 1B | NP_001093087.1 | 1.47E-06 | -3.696608 | chromosome organization |
| chemosensory protein 7 precursor | NP_001037068.1 | 3.78E-15 | -1.73988 | organ development | sex-specific torage-protein 2 precursor | NP_001037590.1 | 0 | -3.967659 | sex-specific protein |
| odorant binding protein | BAH79159.1 | 1.53E-06 | -1.84338 | odorant binding | juvenile hormone binding protein | BAH97095.1 | 1.26E-13 | -4.670473 | juvenile hormone associated |
| prothoracicostatic peptide precursor | NP_001036890.1 | 8.98E-09 | -1.85193 | larval development | diapause associated protein 2 | AFC35301.1 | 3.79E-07 | -9.259743 | diapause associated |
| putative kinesin-associated protein | EHJ67981.1 | 2.24E-11 | -1.88613 | developmental process involved in reproduction | odorant binding protein LOC100307012 precursor | NP_001159621.1 | 3.06E-11 | -9.970106 | odorant binding |
| **Signal transduction** |  |  |  |  |  |  |  |  |  |
| **Name** | **Sequence ID** | **P-Value** | **log2** | **Ontology** | **Name** | **Sequence ID** | **P-Value** | **log2** | **Ontology** |
| alpha-esterase 40 precursor | NP_001116814.1 | 9.85E-09 | 1.2421889 | catalytic activity | putative Sodium/potassium-transporting ATPase subunit beta-2 | EHJ71674.1 | 3.57E-69 | -1.576061 | signal transducer |
| acetylcholinesterase type 1 | ABY50088.1 | 2.15E-06 | 0.583092 | acetylcholine catabolic process | mago nashi | NP_001037630.1 | 2.86E-30 | -1.5961473 | transmembrane receptor protein tyrosine kinase signaling pathway |
| putative Spectrin alpha chain | EHJ74635.1 | 6.79E-08 | -1.029889 | tubulin binding, neurotransmitter secretion | vacuolar ATP synthase subunit E | NP_001040451.1 | 4.02E-17 | -1.06094 | hydrogen ion transmembrane transporter activity |
| hypothetical protein KGM_05953 | EHJ65247.1 | 1.28E-08 | -1.038778 | cation binding,kinase activity | importin-7 | EHJ74083.1 | 1.25E-11 | -1.80689685 | protein transporter activity |
| allatotropin | AAT92286.1 | 1.59E-05 | -1.204225 | hormone activity | receptor for activated protein kinase C RACK 1 isoform 1 | NP_001041703.1 | 1.63E-06 | -2.06844979 | signal transducer activity, transferase activity |
| hypothetical protein KGM_03860 | EHJ68552.1 | 1.51E-05 | -1.236309 | intracellular signal transduction | putative BTB/POZ domain-containing protein KCTD5 | EHJ68424.1 | 2.41E-24 | -2.61867875 | potassium channel activity |
| putative NMDA-type glutamate receptor 1 | EHJ78211.1 | 3.46E-07 | -1.243852 | glutamate receptor activity, on channel activity | hypothetical protein KGM_03033 | EHJ65155.1 | 3.53E-08 | -3.22815523 | regulation of synaptic growth at neuromuscular junction |
| putative BTB/POZ domain-containing protein KCTD9 | EHJ73113.1 | 3.91E-06 | -1.414577 | potassium channel activity | vacuolar ATP synthase catalytic subunit A | NP_001091829.1 | 2.69E-06 | -3.64385619 | hydrogen ion transmembrane transporter activity |
| pro-corazonin precursor | NP_001036899.1 | 4.40E-77 | -1.486792 | hormone activity | putative ceramidase | EHJ63845.1 | 2.69E-06 | -3.64385619 | hydrolase activity, |
| H+ transporting ATP synthase gamma subunit | NP_001040428.1 | 0.257794 | -0.14823 | hydrogen ion transmembrane transporter activity |  |  |  |  |  |
| **Immune response** |  |  |  |  |  |  |  |  |  |
| **Name** | **Sequence ID** | **P-Value** | **log2** | **Ontology** | **Name** | **Sequence ID** | **P-Value** | **log2** | **Ontology** |
| scavenger receptor type C precursor | NP_001128387.1 | 5.09E-09 | 2.2064509 | transmembrane receptor activity | apolipophorin III precursor | NP_001037078.1 | 9.16E-36 | -1.9372325 | binding,defense response to bacterium |
| putative Lysosomal Pro-X carboxypeptidase | EHJ65099.1 | 3.99E-07 | 1.7380013 | hydrolase activity | cuticular protein glycine-rich 10 precursor | NP_001166792.1 | 0.000116952 | -1.98750906 | immune response |
| serine protease 7 precursor | NP_001040537.1 | 1.14E-09 | -1.089256 | immune response | low molecular 30 kDa lipoprotein PBMHP-6 precursor | NP_001037486.1 | 2.90E-279 | -2.10476269 | immune response |
| ATPase inhibitor-like protein | NP_001091821.1 | 1.24E-21 | -1.24119 | enzyme regulator activity | ornithine decarboxylase antizyme 1 | NP_001037028.1 | 9.32E-22 | -2.47858616 | enzyme inhibitor activity |
| serine protease inhibitor 5 precursor | NP_001037205.1 | 3.93E-16 | -1.263185 | endopeptidase inhibitor activity | glutathione S-transferase sigma 1 | NP_001037077.1 | 8.74E-19 | -3.23530451 | transferase activity |
| serine hydroxymethyltransferase | NP_001040279.1 | 1.38E-20 | -1.505673 | hydroxymethyl-, formyl- and related transferase activity | 30kDa protein | ADQ89805.1 | 3.24E-47 | -3.6367635 | immune response |
| putative tumor suppressing subtransferable candidate 1 | EHJ68341.1 | 1.21E-06 | -1.694991 | immune response | fungal protease inhibitor F precursor | NP_001037532.1 | 1.93E-07 | -9.32418055 | fungal protease inhibitor |
| putative thymus-specific serine protease | EHJ67826.1 | 3.76E-05 | -1.733901 | immune response |  |  |  |  |  |
| **Cell cycle** |  |  |  |  |  |  |  |  |  |
| **Name** | **Sequence ID** | **P-Value** | **log2** | **Ontology** | **Name** | **Sequence ID** | **P-Value** | **log2** | **Ontology** |
| hypothetical protein KGM_09809 | EHJ73775.1 | 4.26E-20 | 1.4610614 | cell cycle | meiotic recombination 11 | NP_001036845.1 | 2.31E-14 | -1.64659779 | transition metal ion binding |
| mitosis protein dim1 | EHJ76331.1 | 1.25E-06 | -1.028447 | binding, M phase of mitotic cell cycle | hypothetical protein KGM_02129 | EHJ63237.1 | 1.83E-50 | -1.76154551 | G2/M transition DNA damage checkpoint |
| will die slowly | NP_001037087.1 | 7.42E-11 | -1.101597 | G2/M transition DNA damage checkpoint | hypothetical protein KGM_07972 | EHJ70338.1 | 6.11E-06 | -1.80735492 | ATPase activity, coupled,nucleotide binding |
| DNA-directed DNA polymerase alpha 2 | EHJ77759.1 | 2.15E-15 | -1.128868 | Binding,  telomere maintenance | RNA-binding protein lark | NP_001037293.1 | 1.54E-55 | -1.82070563 | transition metal ion binding, M phase of mitotic cell cycle |
| hypothetical protein KGM_18080 | EHJ70264.1 | 1.58E-06 | -1.329218 | G2/M transition DNA damage checkpoint | 26S protease regulatory subunit | XP_001655853.1 | 6.93E-52 | -2.52988795 | nucleoside-triphosphatase activity |
| putative exosome complex exonuclease RRP41 | EHJ75909.1 | 6.30E-06 | -1.482802 | nucleic acid binding | small nuclear ribonucleoprotein G | EHJ71789.1 | 9.58E-85 | -3.89126466 | translation repressor activity |
| **Apoptosis** |  |  |  |  |  |  |  |  |  |
| **Name** | **Sequence ID** | **P-Value** | **log2** | **Ontology** | **Name** | **Sequence ID** | **P-Value** | **log2** | **Ontology** |
| mitochondrial cytochrome c | ACF41193.1 | 4.54E-18 | 0.098546 | electron carrier activity | hypothetical protein KGM_13696 | EHJ66180.1 | 1.64E-97 | -2.62134046 | hydrogen ion transmembrane transporter activity |
| putative ATP-dependent RNA helicase abstrakt | EHJ74551.1 | 2.39E-09 | -2.020581 | metal ion binding |  |  |  |  |  |
| DEAD box ATP-dependent RNA helicase | EHJ65411.1 | 2.14E-10 | -2.159155 | ATPase activity, coupled | cytochrome c oxidase polypeptide IV | NP_001073120.1 | 1.20E-128 | -3.09786794 | heme-copper terminal oxidase activity |
