# Supplementary material for: Molecular Mechanisms of Reduced Nerve Toxicity by Titanium Dioxide Nanoparticles in the Phoxim-Exposed Brain of Bombyx mori
Source: PLoS One. 2014 Jun 27;9(6):e101062. doi: 10.1371/journal.pone.0101062 (PMC4074129; doi:10.1371/journal.pone.0101062)
Supplement: Table S4 — Primer pairs for qRT-PCR in the gene expression analysis. (DOC) [file pone.0101062.s007.doc]

**Table S4** Primer pairs for qRT-PCR in the gene expression analysis

| Gene name | Primer sequence (5’-3’) | Length of Product (bp) |
| --- | --- | --- |
| *Actin3* | F: CGGCTACTCGTTCACTACC | 147 |
|  | R: CCGTCGGGAAGTTCGTAAG |  |
| *acetylcholinesterase type 1 (ace1)* | CTATGGGCGATTTGAGG | 204 |
|  | GACGTGGTCGAGGTGTC |  |
| *H+ transporting ATP synthase* | AGTCGTGGCGGTGGTTT | 231 |
|  | GGGTCATCTTCGGGAGG |  |
| *vacuolar ATP synthase* | TGCCAAAGGACACCAAA | 129 |
|  | GCAGGGACTCCACCAGA |  |
| *superoxide dismutase (SOD)* | ATGGTGGTCCCAGTTCTGC | 172 |
|  | CCCAAGTCATCAGGGTCAG |  |
| *thiol peroxiredoxin (TPx)* | ACTACGGAGTGCTGGACG | 217 |
|  | TCGGGCTTGATGGTCTT |  |
| *saposin-like (Bm109)* | F: AACGCTACGCCTCACTG | 170 |
|  | R: AACTTGCACGAACCCTC |  |
| *BmIap* | F: AAAACCCGAGGAACTGG | 105 |
|  | R: TCATCGCTTTCCCAATC |  |
| *caspase-9* | F:GAGTGCGTGTTCCTGGTGGTGT | 155 |
|  | R:TTCGGGAGGTCCGTGAAGTTGG |  |
| *caspase-3* | F: AGTTTCGGTCATCTGCTTTAC | 201 |
|  | R: CATTCGGACTTCCTCTTCTTA |  |
| *cytochrome c* | F: CACTGTTGAAGCTGGTGGC | 243 |
|  | R: TTGAGATAGGCAATAAGGT |  |
